# The Wells–Riley Model Revisited II: Parameter Uncertainty and Population Heterogeneity

**DOI:** 10.1111/risa.70272

**Published:** 2026-07-16

**Authors:** Marcus Marshall, Alexander J. Edwards, Dominique Pinnell, Marco‐Felipe King, Catherine J. Noakes, Nazif Elaldi, Grant Lythe, Martín López‐García

**Affiliations:** ^1^ School of Mathematics University of Leeds Leeds UK; ^2^ School of Chemistry University of Bristol Bristol UK; ^3^ School of Civil Engineering University of Leeds Leeds UK; ^4^ Department of İnfectious Diseases and Clinical Microbiology Sivas Cumhuriyet University Sivas Turkey

**Keywords:** airborne transmission, infection risk, parameter uncertainty, Wells–Riley model

## Abstract

In this work, we revisit the Wells–Riley model, which has been widely used to estimate airborne infection risk in indoor settings. In particular, we consider a probabilistic (i.e., “stochastic”) framework of the Wells–Riley model which allows one to quantify infection risk in terms of the per‐capita probability of infection for each susceptible individual, as well as the probability distribution of the number of infections (here referred to as “exposures”) during the indoor interaction. Directly extending the work by Edwards, King, Noakes et al. (2024), we consider here the situation where the main parameters in the Wells–Riley model (namely, the quanta generation rate q, the ventilation rate Q, the number of infectors I, or the duration of the indoor interaction T) may be random or uncertain. We show how, in this case, the per‐capita infection risk $P_{infection}$ becomes a random variable between 0 and 1, and compute its density function under some parametric assumptions. This allows for a comprehensive analytical quantification of uncertainty when dealing with heterogeneous populations, uncertain environmental conditions, or stochastic human behavior. Our results reveal that infection risk can vary significantly depending on the distribution and variability of model parameters. In particular, using mean parameter values in the classical Wells–Riley model can lead to systematic inaccuracies: Uncertainty in q, T, or b leads to infection risk overestimation, while environmental stochasticity (i.e., uncertainty in ventilation or removal rates) can lead to infection risk underestimation. We also investigate which parameter mainly drives the uncertainty in infection risk when two model parameters are simultaneously random.

## 1 Introduction

Airborne transmission is a common mode of infection for various pathogens. It played a particularly significant role in the spread of COVID‐19 during the pandemic that began in early 2020 (Karia et al. 2020). This type of transmission occurs when a susceptible host breathes in small droplets or aerosols laden with an infectious pathogen, usually produced by an infector due to breathing, talking, coughing, sneezing, or during aerosol‐generating procedures. Unlike other routes of infection, such as fomite transmission which is driven by direct contact (e.g., touch) with a contaminated individual or surface, airborne transmission may infect individuals indirectly (e.g., shared air within indoor settings).

Long‐range airborne transmission can be mitigated using ventilation strategies (Qian and Zheng 2018) and through the use of personal protective equipment (PPE) such as masks (Wang et al. 2021). During the early stages of the COVID‐19 pandemic, a limited availability of medical masks resulted in illness among the frontline healthcare workforce (The Lancet 2020). This led to staff shortages and put a strain on healthcare services worldwide (Xu et al. 2020). It is therefore important to understand and assess the risk of airborne transmission particularly in healthcare settings. On the other hand, it is also important to analyze transmission dynamics in non‐healthcare settings, especially in the case of large‐scale or super‐spreading events, which can play a significant role in accelerating population‐level transmission during an epidemic. A paradigmatic example is the Skagit County Choir rehearsal outbreak in Washington, USA, during 2020, where an index case with COVID‐19 was thought to have infected 53 of the 61 attendees, resulting in two deaths (Hamner 2020). The high number of exposures was likely linked to poor ventilation (Miller et al. 2021) and the fact that individuals were singing, which is known to increase the volume of exhaled aerosols (Schijven et al. 2021). This highlights the importance of understanding environmental and behavioral factors affecting airborne transmission risk.

To systematically evaluate infection risk, the Quantitative Microbial Risk Assessment (QMRA) framework can be used. We refer the reader to Haas et al. (2014) for a comprehensive and foundational overview of this methodology. QMRA aims to identify risk factors, and to link pathogen exposure to the probability of infection through the use of mathematical modeling. The use of mathematical models helps to identify the main factors contributing to infection risk, which may inform infection control strategies. Different models can be developed for various routes of transmission or scenarios. For example, QMRA models have been proposed for waterborne exposure (Haas et al. 1993; Cummins et al. 2010; World Health Organization 2016; Doménech et al. 2022; Paraskevopoulos et al. 2024), fomite transmission (Nicas and Jones 2009; Sze‐To et al. 2014; King et al. 2022; Abney et al. 2024; Bate et al. 2024), and airborne transmission (Chen et al. 2006; Denpetkul et al. 2022; Miller et al. 2022; Bate et al. 2024).

A particular QMRA framework to quantify airborne transmission risk is the Wells–Riley model. The Wells–Riley model uses the concept of a *quantum* of infectious pathogen, which was defined by Wells (1955) as the amount of infectious pathogen required to infect a susceptible host upon inhalation. The model was formalized in its classical form by Riley et al. (1978), where it was applied to a case study involving a Measles outbreak within a Florida elementary school. The classical Wells–Riley model assumes well‐mixed air, a steady‐state concentration of pathogen in the air, and a precise knowledge of individual (breathing rate, infectiousness), behavioral (duration of the indoor interaction) and environmental (ventilation) conditions, in terms of specific values of model parameters. Extensions of the Wells–Riley model have been proposed to address some of its main limitations, such as spatial models linking Computational Fluid Dynamics approaches to the Wells–Riley model (Qian et al. 2009; Gupta et al. 2012), adaptations to consider a transient concentration of pathogen in the air over time (Gammaitoni and Nucci 1997), or an extended Wells–Riley model to leverage ${\mathrm{CO}}_{2}$ measurements (Rudnick and Milton 2003). In many real scenarios, it is challenging to determine precise values for the parameters of the Wells–Riley model, due to biological heterogeneity, stochastic human behavior, fluctuating environmental conditions, or due to insufficient/incomplete observations. For example, emission rates or viral loads of individuals within a population may vary due to stage of infection, age, respiratory activity, and other behavioral or physiological factors (Buonanno, Stabile, et al. 2020; Jones, Biele, et al. 2021; Euser et al. 2021; Jones et al. 2024; Aganovic et al. 2024; Sender et al. 2022; Schijven et al. 2021; Mikszewski et al. 2022); removal rates may vary substantially with changing weather, building operation, or room‐level factors (Nazaroff 2021; Edwards, King, López‐García, et al. 2024; Jones, Sharpe, et al. 2021; Henriques et al. 2025; Jones et al. 2025); the duration of indoor interactions is often uncertain or only partially observable (King et al. 2021; Edwards, King, Noakes, et al. 2024). Furthermore, behavioral patterns such as mask‐wearing, movement within indoor environments, and variable contact durations introduce further heterogeneity (King et al. 2021; Huang et al. 2022). At the group level, this uncertainty can significantly affect infection risk estimates, producing highly skewed distributions of secondary infections even under identical environmental conditions (Iddon et al. 2022). They are often difficult to capture using fixed, deterministic parameter values.

Recent work has taken substantial steps to incorporate such uncertainty into airborne infection risk modeling. Monte Carlo and numerical approaches have been used to propagate variability in emission rates, breathing rates, removal processes, prevalence, and occupancy (Bate et al. 2024; Henriques et al. 2025; Iddon et al. 2022; Jones, Sharpe, et al. 2021; Jones et al. 2025; Edwards et al. 2026). Other approaches have examined the role of uncertainty in viral load and emission using quanta‐independent optimization methods (Aganovic et al. 2024). These frameworks have informed building‐design and mitigation guidance, including ASHRAE Standard 241 (Jones et al. 2025), by explicitly embedding parametric uncertainty into their risk‐assessment methodologies. Together, these studies highlight that uncertainty is not an auxiliary component but a central feature of airborne transmission modeling, and that robust infection risk assessment requires methods capable of representing full distributions rather than only mean values.

This study proposes a stochastic, analytical framework to incorporate uncertainty into the Wells–Riley model, extending work previously developed by Nicas (1996) and more recently by Edwards, King, Noakes, et al. (2024). Nicas (1996) incorporated uncertainty into dose–response models by analyzing Beta‐ and Gamma‐distributed doses, while Edwards, King, Noakes, et al. (2024) considered exponentially or Erlang‐distributed quanta emission rates or exposure durations when revisiting the Wells–Riley model. Here, we extend this line of work by deriving analytic expressions for the *full* probability distribution of the per‐capita infection risk when one or two model parameters are uncertain, rather than focusing solely on average risk or relying on Monte Carlo simulation. By treating key parameters as random variables, the per‐capita infection risk itself becomes a random variable in (0,1) with a well‐defined density. Specifically, we consider Gamma‐distributed parameters, which provide the flexibility to model unimodal non‐negative distributions while retaining analytical tractability, and derive closed‐form density functions as well as mean infection risks and exposure distributions. Our results extend those of Nicas (1996) and Edwards, King, Noakes, et al. (2024) by analyzing infection risk under broader classes of random parameters, considering pairs of parameters simultaneously, and incorporating random ventilation (a scenario not addressed in Edwards, King, Noakes, et al. 2024). This framework enables an analytically comprehensive characterization of how uncertainty in emission rates, exposure times, and environmental conditions shapes the distribution of infection risk across different scenarios.

The rest of this paper is organized as follows: In Section 2, we introduce the classical Wells–Riley model and present its stochastic formulation, while incorporating uncertainty into model parameters (both in individual parameters and in pairs); the aim is to compute the density function of the per‐capita infection risk, as well as the probability of observing exactly n exposures. In Section 3, we illustrate our analytical results by means of two case studies to investigate the impact of parameter uncertainty on infection risk. Section 4 contains a final discussion of our approach and findings.

## 2 Methodology

The Wells–Riley model (Wells 1955; Riley et al. 1978) has been widely used to estimate airborne transmission risk during indoor interactions. In particular, for an indoor interaction between S susceptible and I infectious individuals during [0,T], the Wells–Riley model estimates the *per‐capita* infection risk (i.e., the probability of infection for each susceptible individual) as

(1) $$\begin{array}{ccc} P_{infection} & = & 1-e^{-\frac{Ibq}{Q}T}, \end{array}$$

where b is the pulmonary (breathing) rate [$m^{3}\cdot h^{-1}$], q is the quanta emission rate [$\mathrm{quanta}\cdot h^{-1}$], and Q is the ventilation rate [$m^{3}\cdot h^{-1}$]. In this equation, the ventilation rate Q can be replaced by a more general loss parameter which may account for pathogen decay or deposition; see Edwards, King, Noakes, et al. (2024) and Miller et al. (2021). It has recently been shown by Edwards, King, Noakes, et al. (2024) that, since infection events occur independently of each other, the number of infections (which we will synonymously refer to as “exposures” since it is typically the case that these individuals become infected but not infectious during [0,T], since T represents the duration of the indoor interaction which is typically in the order of minutes or hours rather than days) follows a binomial distribution

$$\begin{array}{c} E∼\text{Binomial}(S,P_{\mathrm{infe}\mathrm{ction}}) \end{array}$$

so that the probability of observing exactly n exposures during [0,T] is given by

P(E=n)=SnPinfectionn(1−Pinfection)S−n,n∈{0,1,⋯,S}.

The main assumptions behind the classical Wells–Riley model are:

- The air is well mixed, so that the concentration of pathogen in the air is spatially homogeneous.
- The concentration of pathogen in the air is in steady state during [0,T], neglecting any transient dynamics before this steady‐state concentration is actually reached. A transient version of the model was proposed by Gammaitoni and Nucci (1997); see Edwards, King, Noakes, et al. (2024) for details.
- All individuals behave homogeneously, remain in the indoor environment for the duration of the interaction [0,T], and the model parameters (I, b, q, T, and Q) are known and fixed.

As described in Section 1, recent work in the literature has aimed to address some of these limitations. In particular, numerical approaches or Monte Carlo simulations have been leveraged to incorporate parametric uncertainty and population heterogeneity. On the other hand, a stochastic, mathematical formulation has been recently proposed by Edwards, King, Noakes, et al. (2024) when revisiting the Wells–Riley model, which also addresses some of these points. In particular, Edwards, King, Noakes, et al. (2024) considered the following:

- The situation where the infector(s) leave(s) at time T, but the susceptible individuals remain in the room until T+t for some t≥0.
- The scenario where either b, q, or T are random parameters, following either an Exponential or Erlang distribution. This could represent situations where there is population heterogeneity or to incorporate stochastic human behavior. Analytic results for the expected per‐capita probability of infection and the number of exposures were obtained.

It is interesting to note, however, that if a parameter in {I,b,q,T,Q} is considered to be random, for example, due to uncertainty, the per‐capita infection risk $P_{infection}$ given by Equation (1) is also a random variable, defined in (0,1) (i.e., representing the possible values that $P_{infection}$ can take). That is, if a parameter such as q is random (i.e., infectiousness is unknown/stochastic), and follows a particular probability distribution, the per‐capita infection risk for each susceptible individual, $P_{infection}$, would be uncertain and depend on the specific value of the random parameter q during the specific interaction. For each (random) value of q, $P_{infection}$ would take a different value in (0,1). Thus, Edwards, King, Noakes, et al. (2024) provided an expected (i.e., average) value of this infection risk probability, $E[P_{infection}]$, when either b, q, or T are exponentially‐ or Erlang‐distributed. Here, we propose to go beyond this approach and better capture the uncertainty in infection risk by considering $P_{infection}$ as a random variable in (0,1) and calculating its density function (instead of just its expected value). We consider here Gamma‐distributed parameters, going beyond Exponential or Erlang distributions. The Exponential distribution is obtained by setting the Gamma shape parameter to 1, whereas the Erlang distribution represents a Gamma distribution with integer shape parameter. Gamma distributions yield better parametric flexibility when modeling uni‐modal parametric regimes in the non‐negative line, while allowing for some analytical tractability in Section 2. We note that multimodal regimes can also be considered through linear combinations of Gamma distributions (see Section 2.1.1 and Appendix A1.1). It may be possible to extend some of our results to alternative distributions, as discussed in Section 4. We also consider the possibility of up to two parameters being simultaneously unknown/uncertain rather than just one.

From now on, we classify parameters in the Wells–Riley model as *population‐related* (i.e., mainly related to the individuals involved in the interaction and their behavior: I, b, q, T) or *environmental* (Q). In Subsection 2.1, we present equations which define the density function and mean value of the per‐capita probability of infection $P_{infection}$, as well as the probability of observing exactly n exposures P(E=n) when we have a single, random population‐related parameter. Subsection 2.2 contains similar results when focusing on the environmental parameter Q, whereas in Subsection 2.3, we consider pairs of simultaneously random parameters.

### 2.1 Uncertainty in Population‐Related Parameters

We consider here uncertainty in one of the population‐related parameters {I,b,q,T}. Since these appear in the numerator of the exponential function in the per‐capita infection risk probability in Equation (1), the analysis related to each of these parameters is completely symmetric, with the only difference being that I takes discrete values whereas parameters {b,q,T} take continuous ones.

#### 2.1.1 Uncertainty in Breathing Rate b, Quanta Emission Rate q, or Exposure Time T

We provide here results for the case where the quanta generation rate q is random. Since exactly the same arguments apply to parameters b and T, analogous results are omitted here. Uncertainty in q might be due to uncertainty in estimating its precise value, or alternatively represent population heterogeneity where different individuals in the population might have different quanta generation rates. In particular, we consider the situation where the quanta generation rate follows a Gamma distribution

$$\begin{array}{c} q∼\text{Gamma}(k,\lambda ), \end{array}$$

with shape parameter k>0 and rate parameter λ>0. That is, q has probability density function

$$\begin{array}{cc} f_{q}\left(x\right)= & \frac{\lambda^{k}x^{k-1}\exp\left(-\lambda x\right)}{\Gamma (k)},\;x>0, \end{array}$$

where Γ(k) is the Gamma function. We note that the particular case where k is a positive integer leads to an Exponential distribution, q∼Exp(λ), if k=1 or Erlang distribution, q∼Erlang(k,λ), if k=2,3,⋯. As discussed above, if q is random the per‐capita infection risk

$$\begin{array}{ccc} P_{infection} & = & 1-e^{-\frac{Ibq}{Q}T}, \end{array}$$

is also random. That is, $P_{infection}$ is a random variable which can take different values in (0,1) depending on the value of q that happens to “occur” during a specific indoor interaction. The aim here is to capture the uncertainty in $P_{infection}$ caused by the randomness of q, by computing the probability density function of the random variable $P_{infection}$. For q∼Gamma(k,λ), this can be calculated as (see Appendix A1.1)

(2) $$\begin{array}{ccc} f_{P_{infection}}\left(p\right) & = & {\left(\frac{\lambda Q}{IbT}\right)}^{k}\frac{{\left[-\ln\left(1-p\right)\right]}^{k-1}{\left(1-p\right)}^{\frac{\lambda Q}{IbT}-1}}{\Gamma (k)}, \end{array}$$

such that p∈(0,1). This density function allows one to estimate how likely different values of $P_{infection}$ are within the interval (0,1), based on the distribution of q. We note that this expression is consistent with an analogous result obtained by Nicas (1996) in the context of dose–response models, when considering a Gamma‐distributed dose. As expected, the density function in Equation (2) depends on parameters {I,b,Q,T} but does not depend on q itself, since it is not a fixed parameter value here but a random parameter instead. Thus, the density function of the per‐capita infection risk probability depends instead on the extra parameters (k,λ) governing the distribution of q. From this, the “expected” or “average” per‐capita infection risk is (see Appendix A1.1)

(3) $$\begin{array}{c} E\left[P_{infection}\right]=1-{\left(\frac{\lambda }{\lambda +\frac{IbT}{Q}}\right)}^{k}. \end{array}$$

This expression provides an *exact* average infection risk while accounting for the randomness or uncertainty in parameter q, whereas the full infection risk profile is captured by the density function of $P_{infection}$ given by Equation (2).

It is interesting to explore what would happen if one considered the average quanta generation rate $\bar{q}=E[\text{Gamma}(k,\lambda )]=k/\lambda$ as a representative parameter value for q, and plugged it into the classical Wells–Riley approach to obtain a potentially representative per‐capita infection risk probability

$$\begin{array}{ccc} {\bar{P}}_{infection}^{WR} & = & 1-e^{-\frac{IbT}{Q}\bar{q}}\;=\;1-e^{-\frac{IbT}{Q}\frac{k}{\lambda }}. \end{array}$$

This is a typical approach in the literature when implementing the classical Wells–Riley framework to estimate infection risk under parameter uncertainty. Interestingly, it can be shown that ${\bar{P}}_{infection}^{WR}$ will always overestimate the “true” mean $E[P_{infection}]$ in Equation (3) (which represents the *exact* average infection risk probability which fully accounts for the randomness in q); that is,

$$\begin{array}{c} {\bar{P}}_{infection}^{WR}>E\left[P_{infection}\right] \end{array}$$

for all parameter regimes (I,b,T,Q,k,λ). In fact, this holds for any general, positive‐valued distribution of q (equivalently, of b or T), and not just a Gamma distribution, since this relies only upon the concavity of the Wells–Riley equation and the use of Jensen's inequality; see Appendix A5.1.

**FIGURE 1:**
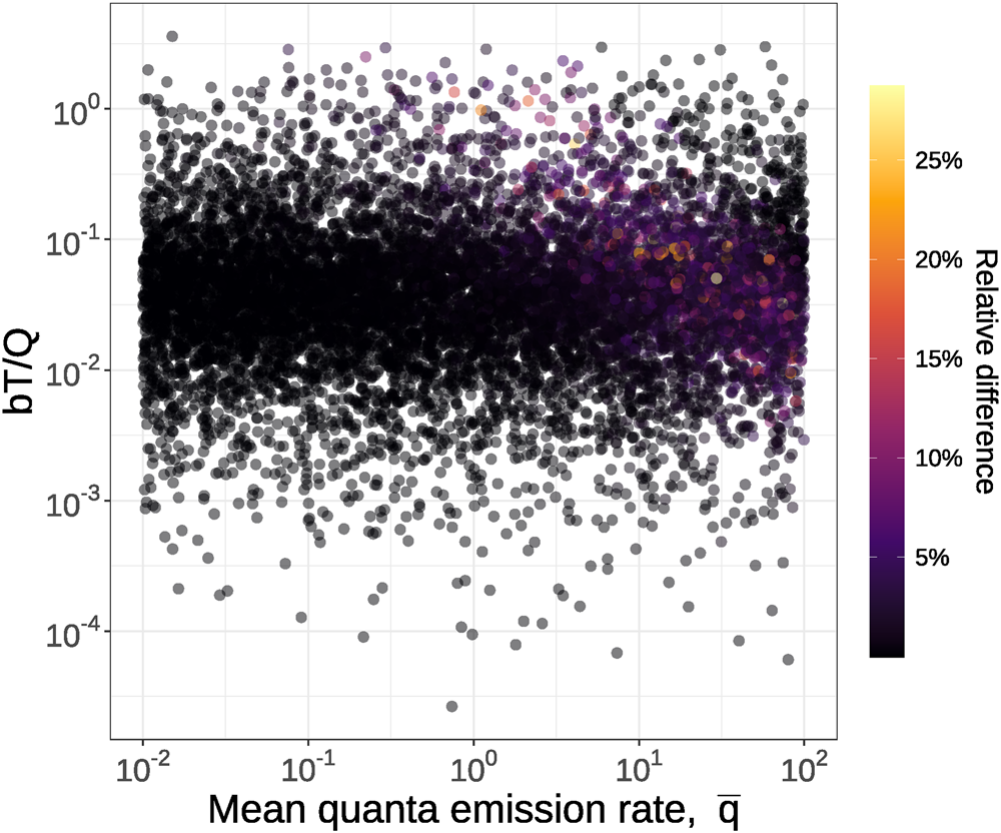
Scatter plot for the relative difference between ${\bar{P}}_{infection}^{WR}$ (classical Wells–Riley approach with constant, average quanta generation rate $\bar{q}=k/\lambda$) and the *exact* analytical mean infection risk $E[P_{infection}]$ in Equation (3), versus $\bar{q}$ and $\frac{bT}{Q}$. The shape and rate parameters (k,λ) of the Gamma distribution of q, as well as parameters (b,T,Q), are randomly sampled within relatively wide ranges. In particular, $\log_{10}\left(\bar{q}\right)\in \left[-2,2\right]$, k∈[1,20] (such that we set $\lambda =k/\bar{q}$ and explore $\bar{q}\in \left[0.01,100\right]\;\mathrm{quanta}\cdot h^{-1}$), b∈[0.2,0.6] $m^{3}\cdot h^{-1}$, $Q\in \left[10,1000\right]\;m^{3}\cdot h^{-1}$ (approximately ACH∈[0.1,10] for a volume of $V\approx 100\;m^{3}$ (Edwards 2024)), and T∈[0.1,100]h.

For illustrative purposes, we plot in Figure 1 the relative difference,

$$\text{Relative difference}\left(\%\right)=\frac{{\bar{P}}_{infection}^{WR}-E\left[P_{infection}\right]}{E[P_{infection}]}\times 100,$$

between the classical approach, ${\bar{P}}_{infection}^{WR}$, and the analytical mean infection risk, $E[P_{infection}]$ in Equation (3), for parameter values (k,λ,b,T,Q) randomly sampled within relatively wide ranges, and average quanta generation rates in $\bar{q}\in \left[0.01,100\right]$ $\mathrm{quanta}\cdot h^{-1}$ to represent a wide range of emission rates. As expected, strictly positive relative differences correspond to infection risk overestimation by the classical approach versus its exact average counterpart. The plot suggests that parametric choices of (b,T,Q) leading to relatively large values of $e^{\frac{bT}{Q}}$, combined with sufficiently high $\bar{q}$, lead to larger relative differences. In particular, we observe overestimations of the analytical mean by the classical approach on the magnitude of 5%−27% for a localized “strip” in the upper right region in Figure 1. Below and to the left of this strip, as well as above and to the right, we observe little to no relative difference. We note that these two darker regions correspond to a combination of low (below the strip) and high (above the strip) values of bT/Q and $\bar{q}$, resulting in both estimates, ${\bar{P}}_{infection}^{WR}$ and $E[P_{infection}]$, tending toward zero or one. The region of bT/Q at which overestimations occur are dependent on the value of $\bar{q}$. It is also interesting to note that nearby dots in Figure 1 can still have different colors indicating different relative differences. This is due to the particular distribution of q corresponding to those dots, which is determined by the specific values of (k,λ). Wider Gamma distributions around $\bar{q}$ (obtained for smaller values of k, with $\lambda =k/\bar{q}$) translate to higher skewness and variance, representing higher uncertainty around $\bar{q}$. Overall, our results suggest that larger variance and skewness of q lead to larger relative differences between ${\bar{P}}_{infection}^{WR}$ and $E[P_{infection}]$. In particular, the classical approach with average $\bar{q}$ does not appropriately account for the possibility of small values of q happening with positive probability, which would lead to low infection risk in reality; thus, ${\bar{P}}_{infection}^{WR}$ can overestimate the real average infection risk in these situations, better captured by $E[P_{infection}]$. It should be mentioned that for the values considered in Figure 1, relative differences for larger $\bar{q}$ and bT/Q correspond to larger absolute differences in the infection risk estimates. The relative overestimations for different values of $\bar{q}$ and bT/Q have been analyzed in further detail in Appendix A5.1. Since these infection risk estimates may be used in practice to propose or quantify the impact of specific mitigation strategies (Gammaitoni and Nucci 1997; Chen et al. 2006; Bazant and Bush 2021), our results highlight the importance of accounting for parametric uncertainty when estimating infection risk; in particular, implementing the classical Wells–Riley approach under average quanta generation rates seems to lead to overestimations of up to 27% for the parameter values considered in Figure 1.

Along with the infection risk density and mean infection risk (Equations (2) and (3)), the probability of observing exactly n exposures among S susceptible individuals during [0,T] is (see Appendix A1.1)

(4) P(E=n)=Sn∑i=0n(−1)iniλ(S−n+i)IbTQ+λk,

for n∈{0,1,⋯,S}. We note that Equations (3) and (4) are consistent with those previously derived by Edwards, King, Noakes, et al. (2024), whereas Equation (2) is a novel result which allows one to analytically capture the uncertainty that random q generates on the per‐capita infection risk estimate $P_{infection}$. Random parameters b or T would lead to analogous results to those above, and are thus not reported explicitly here. It is also worth noting that the density function in Equation (2) is consistent with that derived by Nicas (1996) in the context of a dose–response model, where the dose is considered to be random instead of the specific parameters (b,q,T) in the Wells–Riley framework.

Finally, we note that random q might also arise as a result of mitigations, human behavior or population heterogeneity, and our mathematical formulation can be adapted to consider these situations. For example, one may consider that the infector wears a mask with probability $w_{mask}\in \left[0,1\right]$, so that the average quanta generation rate is

$$\begin{array}{ccc} {\overset{-}{q}}_{\mathrm{eff}} & = & q_{\mathrm{no}\mathrm{mask}}(w_{\mathrm{mask}}(1-\eta )+(1-w_{\mathrm{mask}})), \end{array}$$

where η∈(0,1) represents the reduction in quanta generation due to mask filtration efficacy, and $q_{\mathrm{no}\mathrm{mask}}$ is the underlying quanta emission rate without mask‐wearing. For simplicity, we do not consider mask‐wearing by susceptible individuals and focus instead on the impact of mask‐wearing by the infector on infection risk. In this scenario, the Wells–Riley equation effectively becomes (Gammaitoni and Nucci 1997; Bazant and Bush 2021; Rothamer et al. 2021; Huang et al. 2022)

$$\begin{array}{c} P_{\mathrm{infe}\mathrm{ction}}=1-e^{-\frac{\mathrm{Ib}{\overset{-}{q}}_{\mathrm{eff}}T}{Q}}. \end{array}$$

One can still consider uncertainty in the underlying quanta emission rate, $q_{no\;mask}∼\text{Gamma}\left(k,\lambda \right)$, while incorporating stochastic human behavior through probability $w_{mask}$, so that the effective quanta emission rate, $q_{eff}$, is a random variable with density function

$$\begin{array}{ccc} f_{q_{eff}}\left(q\right) & = & w_{mask}\frac{1}{1-\eta }f_{q_{no\;mask}}\left(\frac{q}{1-\eta }\right)+\left(1-w_{mask}\right)f_{q_{no\;mask}}\left(q\right). \end{array}$$

Our results then can be naturally extended so that (see Appendix A1.1)

(5) $$\begin{array}{ccc} f_{P_{infection}}\left(p\right) & = & \frac{{\left(-\ln\left(1-p\right)\right)}^{k-1}}{\Gamma (k)}{\left(\frac{\lambda Q}{IbT}\right)}^{k}(w_{mask}{\left(1-\eta \right)}^{-k}{\left(1-p\right)}^{\frac{\lambda Q}{IbT(1-\eta )}-1} \end{array}$$

(6) $$\begin{array}{ccc} & & +\left(1-w_{mask}\right){\left(1-p\right)}^{\frac{\lambda Q}{IbT}-1}),\;\text{for}\;p\in (0,1),\\ E[P_{infection}] & = & 1-\left[w_{mask}{\left(\frac{\lambda }{\lambda +\frac{IbT}{Q}\left(1-\eta \right)}\right)}^{k}+\left(1-w_{mask}\right){\left(\frac{\lambda }{\lambda +\frac{IbT}{Q}}\right)}^{k}\right], \end{array}$$

(7) P(E=n)=Sn∑i=0n(−1)ini[wmaskλλ+(S−n+i)IbTQ(1−η)k+(1−wmask)λλ+(S−n+i)IbTQk],forn=0,1,⋯,S.

A slightly different application arises when one aims to represent population heterogeneity, so that q∼Gamma(k,λ) is a population‐level description of infectiousness. However, since the term qI in Equation (1) represents the rate of quanta generation by all I infectious individuals during the indoor interaction, these individuals are all still assumed to generate quanta at the same rate q, which can be globally sampled from Gamma(k,λ). This is appropriate when I=1, so that the infector's infectiousness is unknown and sampled from a population‐level distribution. On the other hand, for scenarios where more than one, I>1, infectious individual is assumed to be present within the interaction, it is more appropriate to replace the term qI by $\sum_{i=1}^{I}q_{i}$, where $q_{i}$ represents the quanta generation rate of each individual i, which can be separately sampled from the population‐level distribution Gamma(k,λ), so that $q_{i}∼\text{Gamma}(k,\lambda )$ are independent and identically distributed random variables. Although not the main focus of this paper, we generalize the results above to this situation in Appendix A4.

#### 2.1.2 Disease Prevalence

While it is common to assume that the number of infectors I during the indoor interaction is known, this is not typically the case in reality when trying to assess risk in different settings a priori. In particular, by assuming that a single infector is present in the room (i.e., by setting I=1), the per‐capita probability of infection may well be overestimated, since one of the main factors impacting infection risk is the probability of an infector being present in the room at all. Thus, it may be more appropriate to consider a disease prevalence ρ, understood here as the proportion of individuals in the population being infectious at any given time, so that (assuming independence) the number of infectious individuals in the room is random and follows a binomial distribution

$$\begin{array}{ccc} I & ∼ & \text{Binomial}(N,\rho ), \end{array}$$

where N is the total number of individuals involved in the indoor interaction. The probability mass function of I is given by:

P(I=i)=Niρi(1−ρ)N−i,i∈{0,1,⋯,N}.

Importantly, this incorporates the possibility that there might not be infectors present during the interaction (I=0), which occurs with probability ${\left(1-\rho \right)}^{N}$, or that there might be more than one (I>1). Since I is a discrete random variable with support {0,1,⋯,N}, the per‐capita infection risk $P_{infection}$ is also a discrete random variable on this occasion. Thus, its probability mass function is (see Appendix A1.2)

(8) fPinfection(p)=Niρi(1−ρ)N−i

with possible (positive probability) values $p=1-e^{-\frac{bqT}{Q}i}$ where i∈{0,1,⋯,N}. The expected per‐capita infection risk is (see Appendix A1.2)

(9) $$\begin{array}{c} E\left[P_{infection}\right]=1-{\left[\rho e^{-\frac{bqT}{Q}}+\left(1-\rho \right)\right]}^{N}. \end{array}$$

The probability that there are exactly n exposures is given by (see Appendix A1.2)

(10) P(E=n)=Nn∑i=0N−nN−ni1−e−bqTQine−bqTQi(N−i−n)ρi(1−ρ)N−i,

for n=0,1,⋯,N.

### 2.2 Uncertainty in Ventilation Rate

Here, we consider the situation where the ventilation rate Q is uncertain (i.e., random), for example, in situations where this may depend on external factors (e.g., weather) or human behavior (e.g., windows opening). We consider a Gamma distribution

$$\begin{array}{ccc} Q & ∼ & \text{Gamma}(r,\gamma ), \end{array}$$

with shape parameter r>0 and rate parameter γ>0. Since Q appears in the denominator (instead of the numerator) of the exponent in the per‐capita infection risk probability in Equation (1), the results obtained in Section 2.1 for b, q, and T do not directly apply here. Instead, for Q∼Gamma(r,γ), the density function of the per‐capita infection risk $P_{infection}$ is (see Appendix A2)

(11) $$\begin{array}{c} f_{P_{infection}}\left(p\right)=\frac{{\left(\gamma IbqT\right)}^{r}}{\Gamma (r)}\frac{e^{\frac{\gamma IbqT}{\ln(1-p)}}}{\left(1-p\right){\left[-\ln\left(1-p\right)\right]}^{r+1}},\;p\in \left(0,1\right). \end{array}$$

As a result, the average per‐capita infection risk is given by (see Appendix A2)

(12) $$\begin{array}{c} E\left[P_{infection}\right]=1-\frac{2}{\Gamma (r)}{\left(\gamma IbqT\right)}^{r/2}K_{r}\left(2\sqrt{\gamma IbqT}\right), \end{array}$$

where $K_{\theta }\left(\zeta \right)$ is the modified Bessel function of the second kind (NIST 2025a)

$$\begin{array}{c} K_{\theta }\left(\zeta \right)=\frac{1}{2}{\left(\frac{\zeta }{2}\right)}^{\theta }\int_{0}^{\infty }u^{-(\theta +1)}e^{\left(-u-\frac{\zeta^{2}}{4u}\right)}du. \end{array}$$

Finally, the probability of observing exactly n exposures is (see Appendix A2)

(13) P(E=n)=2Γ(r)Sn∑i=0n(−1)iniair/2Kr2ai

for n=0,1,⋯,S, where $a_{i}=\gamma IbqT\left(S-n+i\right)$. We note that for the special case n=S, the factor (S−n+i) equals 0 when i=0. Since $K_{\theta }\left(\zeta \right)∼2^{\;\theta -1}\Gamma \left(\theta \right)\;\zeta^{-\theta }$ when ζ→0 (NIST 2025b), one has $a_{i}^{r/2}K_{r}\;\left(2\sqrt{a_{i}}\right)\;\to \;\frac{1}{2}\Gamma \left(r\right)$ when (S−n+i)→0 (meaning $a_{i}\to 0$), so that in the i=0 term, one gets $\frac{2}{\Gamma (r)}\times \frac{1}{2}\Gamma \left(r\right)=1$ (since the other elements in the term are also 1).

**FIGURE 2:**
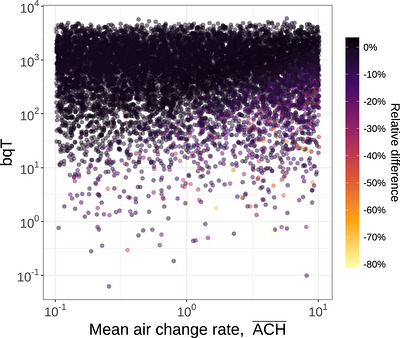
Scatter plot for the relative difference between ${\bar{P}}_{infection}^{WR}$ (classical Wells–Riley approach with constant, average ventilation rate $\bar{Q}=r/\gamma$) and the *exact* analytical mean infection risk $E[P_{infection}]$ in Equation (12), versus $\bar{Q}$ and bqT. The shape and rate parameters (r,γ) of the Gamma distribution of Q, as well as parameters (b,q,T), are randomly sampled within relatively wide ranges. In particular, $\log\left(\bar{Q}\right)\in \left[1,3\right]$, r∈[1,20] (such that we set $\gamma =r/\bar{Q}$ and explore $\bar{ACH}\in \left[0.1,10\right]$ for a room volume V≈100 m 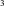 (Edwards 2024)), b∈[0.2,0.6] $m^{3}\cdot h^{-1}$, $q\in \left[0.01,100\right]\;\mathrm{quanta}\cdot h^{-1}$, and T∈[0.1,100]h.

Similar to our analysis in Section 2.1 for random q, we can investigate here the relative difference between the infection risk estimated via the classical Wells–Riley approach

$${\bar{P}}_{\mathrm{infection}}^{WR}=1-e^{-\frac{IbqT}{\bar{Q}}},$$

with average ventilation rate $\bar{Q}=r/\gamma$, and the *exact* analytical average infection risk $E[P_{infection}]$ in Equation (12) (which accounts for the randomness in Q). This relative difference is plotted in Figure 2. While considering random q systematically led to overestimating infection risk under the classical approach (due to Jensen's inequality), the fact that $P_{infection}$ is not necessarily concave on Q means that it is not possible to determine a priori if the relative difference would be positive or negative depending on the particular values of (r,γ,b,q,T). Still, we tend to see infection risk underestimation (i.e., negative relative difference) in the case of random ventilation for most of the parametric regimes considered in Figure 2. This is due to $P_{infection}$ being convex on Q for the majority of parameter values considered. In fact, where there are overestimations (positive relative errors corresponding to black‐colored points), these are very close to zero, and occur for very small ventilation rates combined with high values of bqT, such that both the classical and analytical estimates each tend toward 1. Outside of this specific region, it appears as though the size of the relative difference is largely independent of the specific values of bqT and $\bar{ACH}$. In fact, as was the case for random quanta emission, it increases with higher variance and positive skewness of the Gamma distribution of the ventilation rate (something we cannot see by plotting against $\bar{ACH}$ alone). The magnitude of these inaccuracies are far larger in the case of random Q compared to that of random q (relative underestimations of 80% vs. overestimations of 25% of the analytical $E[P_{infection}]$, see Figures 1 and 2). This is because the infection risk does not vary so significantly in the region of $\bar{q}$ as it does for $\bar{Q}$: Variations in Q leading to small ventilation rates produce higher stochasticity in the infection risk than variation in q, since in the case of random Q scenarios with very low ventilation can lead to notably high infection risk. In Appendix A5.2, the size of the relative difference has been investigated further for the parameter space considered here.

Finally, we note that if a particular model parameter has a non‐random component, our results can be easily adapted. For example, we explore in Section 3 the situation where natural ventilation in an indoor environment might be stochastic, whereas mechanical ventilation can be more precisely controlled or estimated. In this situation, one might define the ventilation rate as $Q=Q_{nat}+Q_{mech}$ where $Q_{\mathrm{nat}}∼\text{Gamma}(r,\gamma )$ and $Q_{mech}$ is constant, so that

(14) $$\begin{array}{c} P_{infection}=1-e^{-\frac{IbqT}{Q_{nat}+Q_{mech}}}. \end{array}$$

Our results can be extended to this situation, so that, for example, the probability density function of the infection risk is given as

(15) $$\begin{array}{cc} f_{P_{\mathrm{infection}}}(p)= & \frac{\gamma^{r}{\left(\frac{-\mathrm{IbqT}}{\ln(1-p)}-Q_{\mathrm{mech}}\right)}^{r-1}e^{\frac{\gamma \mathrm{IbqT}}{\ln(1-p)}+\gamma Q_{\mathrm{mech}}}\mathrm{IbqT}}{\Gamma (r)(1-p)(\ln{\left(1-p)\right)}^{2}},\;p\in \left(0,1-e^{-\frac{\mathrm{IbqT}}{Q_{\mathrm{mech}}}}\right); \end{array}$$

see Appendix A2.1 for further details.

### 2.3 Uncertainty in Two Model Parameters

We now consider the situation where two independent parameters are simultaneously random. The aim is to investigate their compounding effect on infection risk. We can consider two distinct cases: Two population‐related parameters are random, or one population‐related parameter is random as well as ventilation (the environmental parameter). Let us start with the former case where two population‐related parameters {b,q,T} are random; say the quanta emission rate q and the exposure time T without any loss of generality (since equivalent arguments would apply to {b,q} or {b,T}). In particular, we consider

$$\begin{array}{ccc} q & ∼ & \text{Gamma}(k,\lambda ),\\ T & ∼ & \text{Gamma}(\ell ,\mu ), \end{array}$$

for shapes k,ℓ>0 and rates λ,μ>0. In this scenario, the density function of the per‐capita infection risk $P_{infection}$ is (see Appendix A3)

(16) $$\begin{array}{cc} f_{P_{infection}}\left(p\right)= & \frac{2{\left(\lambda \mu \right)}^{\frac{k+\ell }{2}}{\left[-\frac{1}{c}\ln\left(1-p\right)\right]}^{\frac{k+\ell }{2}-1}K_{k-\ell }\left(2\sqrt{d\left[-\ln(1-p)\right]}\right)}{c\Gamma (k)\Gamma (\ell )(1-p)} \end{array}$$

with $c=\frac{Ib}{Q}$, $d=\frac{\lambda \mu }{c}$, and for p∈(0,1). The average per‐capita infection risk is given by (see Appendix A3)

(17) $$\begin{array}{cc} E[P_{infection}]= & 1-d^{\ell }U\left(\ell ,\ell -k+1,d\right)=1-d^{k}U\left(k,\;k-\ell +1,d\right), \end{array}$$

where U(a,b,z) is the confluent hypergeometric function of the second kind (NIST 2025c), sometimes referred to as the *Tricomi* function

$$\begin{array}{c} U\left(a,b,z\right)=\frac{1}{\Gamma (a)}\int_{0}^{\infty }e^{-zv}v^{a-1}{\left(1+v\right)}^{b-a-1}dv. \end{array}$$

The probability of observing exactly n exposures is given by (see Appendix A3)

(18) P(E=n)=Sn∑i=0n(−1)iniyiℓU(ℓ,ℓ−k+1,yi)

(19) =Sn∑i=0n(−1)iniyikU(k,k−ℓ+1,yi)

for n=0,⋯,S, where $y_{i}=\frac{d}{\left(S-n+i\right)}$. We note here that when S−n+i=0 we use the limit of the Tricomi function (NIST 2025d) $U\left(a,b,z\right)∼z^{-a}$ when z→∞. Given that i=0 and S=n (where S−n+i=0), the first term in each sum would evaluate to $y_{0}^{\ell }y_{0}^{-\ell }=y_{0}^{k}y_{0}^{-k}=1$. We also note that each of the two forms proposed for $E[P_{infection}]$ and P(E=n) is derived by first solving the underlying double integral either with respect to q or T. The equivalence of these two forms is further validated by Kummer's transformation (NIST 2025e) $U\left(a,b,z\right)=z^{1-b}U\left(1+a-b,2-b,z\right)$.

Finally, it is important to note that some of our results above simplify when one considers an Erlang‐distributed parameter instead of Gamma (i.e., when the shape parameter is an integer). For example, Equations (17)–(19) may be written as finite sums if k∈N (i.e., q is Erlang‐distributed):

(20) E[Pinfection]=1−ed(k−1)!∑i=0k−1k−1i(−1)idi+ℓΓk−ℓ−i,d,

(21) P(E=n)=Sn∑i=0n∑j=0k−1(−1)i+j(k−1)!nik−1jyiℓ+jeyiΓ(k−ℓ−j,yi),

for n=0,⋯,S, where

$$\begin{array}{c} \Gamma \left(s,x\right)=\int_{x}^{\infty }w^{s-1}e^{-w}dw \end{array}$$

is the upper incomplete Gamma function (NIST 2025f). If T is Erlang‐distributed instead of q (i.e., if ℓ∈N instead of k) one gets analogous results omitted here. Further simplified expressions are obtained if both parameters are exponentially distributed, k=ℓ=1, in which case

fPinfection(p)=2d(1−p)K02d−ln(1−p),p∈(0,1),E[Pinfection]=1−dedΓ(0,d),P(E=n)=Sn∑i=0n(−1)iniyieyiΓ(0,yi),

for n=0,⋯,S.

Finally, we consider here the case where one population‐related parameter from {b,q,T} is random alongside the ventilation rate Q. Without loss of generality we consider q to be random, in particular,

$$\begin{array}{ccc} q & ∼ & \text{Gamma}(k,\lambda ),\\ Q & ∼ & \text{Gamma}(r,\gamma ). \end{array}$$

The density function of the per‐capita infection risk $P_{infection}$ is (see Appendix A3)

(22) $$\begin{array}{c} f_{P_{infection}}\left(p\right)=\frac{\Gamma (k+r)}{\Gamma (k)\Gamma (r)}\frac{\lambda^{k}{\left(\gamma IbT\right)}^{r}{\left[-\ln\left(1-p\right)\right]}^{k-1}}{\left(1-p\right){\left(\left[-\ln\left(1-p\right)\right]\lambda +\gamma IbT\right)}^{k+r}} \end{array}$$

for p∈(0,1). The expected per‐capita infection risk is given by (see Appendix A3)

(23) $$\begin{array}{c} E\left[P_{infection}\right]=1-{\left(\frac{\gamma IbT}{\lambda }\right)}^{r}\frac{\Gamma (k+r)}{\Gamma (r)}U\left(k+r,r+1,\frac{\gamma IbT}{\lambda }\right). \end{array}$$

The probability of n exposures is (see Appendix A3)

(24) P(E=n)=Sn∑i=0nni(−1)iαirΓ(k+r)Γ(r)Uk+r,r+1,αi,

for n=0,⋯,S, where $\alpha_{i}=\frac{\gamma IbT}{\lambda }\left(S-n+i\right)$. If k,r∈N, then Equations (23) and (24) simplify to

(25) E[Pinfection]=1−eγIbTλ(r−1)!∑i=0r+k−1r+k−1i(−1)iγIbTλiΓr−i,γIbTλ,

(26) P(E=n)=Sn1(r−1)!∑i=0n∑j=0r+k−1(−1)i+jnir+k−1jαijeαiΓr−j,αi,

for n=0,⋯,S. Additionally, when k=r=1,

fPinfection(p)=λγIbT(1−p)([−ln(1−p)]λ+γIbT)2,p∈(0,1)E[Pinfection]=eγIbTλγIbTλΓ0,γIbTλ,P(E=n)=Sn∑i=0n(−1)ini1−eαiαiΓ(0,αi),

for n=0,⋯,S, where we have simplified Equations (25) and (26), along with the following property (NIST 2025g); if s∈N,

$$\begin{array}{c} \Gamma \left(s,x\right)=\left(s-1\right)!e^{-x}\sum_{i=0}^{s-1}\frac{x^{i}}{i!}. \end{array}$$

If instead s=−n with $n\in N_{0}=\left\{0,1,2,\cdots \right\}$, then (NIST 2025h)

$$\begin{array}{c} \Gamma \left(-n,x\right)=\frac{{\left(-1\right)}^{n}}{n!}\left(\Gamma \left(0,x\right)-e^{-x}\sum_{i=0}^{n-1}\frac{{\left(-1\right)}^{i}i!}{x^{i+1}}\right). \end{array}$$

Each of these may be used to compute values for Equations (20) and (21) and (25) and (26), instead of trying to evaluate multiple incomplete Gamma functions.

## 3 Results

In Section 2, we have extended the Wells–Riley methodology while considering Gamma‐distributed parameters, computing the density function of the per‐capita infection risk probability, its mean, and the probability distribution of the number of exposures. These analytical results are summarized in Table 1, where results for a random quanta emission rate, q, are analogous to those for a random breathing rate, b, or exposure time, T. In this section, we aim to explore the impact that uncertainty in these parameter values has on infection risk analytically, by considering Gamma distributions for some of these parameters. While other distributions may lead to slightly better representations, our aim is not to find the best distribution in each scenario, but to illustrate instead our analytical results in Section 2, while showing the impact of incorporating uncertainty in parameter values. We discuss how some of our results may be generalized to alternative distributions in Section 4. In this section, we consider different parametric regimes inspired by scenarios related to healthcare and hospitality. First, we outline in Section 3.1 some of these parametric choices.

**TABLE 1:** Table summarizing analytical results from Section 2.

Scenarios	Infection probability density, $f_{P_{\mathrm{infe}\mathrm{ction}}}\left(p\right)$, for p∈(0,1) unless otherwise stated	Expected infection probability, $E[P_{\mathrm{infection}}]$	Probability of n exposures, P(E=n), for n=0,⋯,S unless otherwise stated
Random quanta emission rate, q∼Gamma(k,λ). Constant (I,b,T,Q).	${\left(\frac{\lambda Q}{IbT}\right)}^{k}\frac{{\left[-\ln\left(1-p\right)\right]}^{k-1}{\left(1-p\right)}^{\frac{\lambda Q}{IbT}-1}}{\Gamma (k)}$	$1-{\left(\frac{\lambda }{\lambda +\frac{IbT}{Q}}\right)}^{k}$	Sn∑i=0n(−1)iniλ(S−n+i)IbTQ+λk
Random ventilation rate, Q∼Gamma(r,γ). Constant (I,b,T,q).	$\frac{{\left(\gamma IbqT\right)}^{r}}{\Gamma (r)}\frac{e^{\frac{\gamma IbqT}{\ln(1-p)}}}{\left(1-p\right){\left[-\ln\left(1-p\right)\right]}^{r+1}}$	$1-\frac{2}{\Gamma (r)}{\left(\gamma IbqT\right)}^{r/2}K_{r}\left(2\sqrt{\gamma IbqT}\right)$	2Γ(r)Sn∑i=0n(−1)iniair/2Kr2ai, where $a_{i}=\gamma IbqT\left(S-n+i\right)$
Random quanta emission rate, q∼Gamma(k,λ). and exposure time T∼Gamma(ℓ,μ). Constant (I,b,Q).	$\frac{2{\left(\lambda \mu \right)}^{\frac{k+\ell }{2}}{\left[-\frac{1}{c}\ln\left(1-p\right)\right]}^{\frac{k+\ell }{2}-1}K_{k-\ell }\left(2\sqrt{d\left[-\ln(1-p)\right]}\right)}{c\Gamma (k)\Gamma (\ell )(1-p)}$, where $c=\frac{Ib}{Q}$, $d=\frac{\lambda \mu }{c}$	$1-d^{\ell }U\left(\ell ,\ell -k+1,d\right)=1-d^{k}U\left(k,\;k-\ell +1,d\right)$	Sn∑i=0n(−1)iniyiℓU(ℓ,ℓ−k+1,yi) =Sn∑i=0n(−1)iniyikU(k,k−ℓ+1,yi), where $y_{i}=\frac{d}{\left(S-n+i\right)}$
Random quanta emission rate, q∼Gamma(k,λ). and ventilation rate Q∼Gamma(r,γ). Constant (I,b,T).	$\frac{\Gamma (k+r)}{\Gamma (k)\Gamma (r)}\frac{\lambda^{k}{\left(\gamma IbT\right)}^{r}{\left[-\ln\left(1-p\right)\right]}^{k-1}}{\left(1-p\right){\left(\left[-\ln\left(1-p\right)\right]\lambda +\gamma IbT\right)}^{k+r}}$	$1-{\left(\frac{\gamma IbT}{\lambda }\right)}^{r}\frac{\Gamma (k+r)}{\Gamma (r)}U\left(k+r,r+1,\frac{\gamma IbT}{\lambda }\right)$	Sn∑i=0nni(−1)iαirΓ(k+r)Γ(r)Uk+r,r+1,αi, where $\alpha_{i}=\frac{\gamma IbT}{\lambda }\left(S-n+i\right)$
Random number of infectors, I∼Binomial(N,ρ). Constant (q,b,T,Q).	Niρi(1−ρ)N−i,ifp=1−e−bqTQi,0,otherwise, for i=0,⋯,N	$1-{\left[\rho e^{-\frac{bqT}{Q}}+\left(1-\rho \right)\right]}^{N}$	Nn∑i=0N−nN−niρi(1−ρ)N−i $\times {\left(1-e^{-\;\frac{bqT}{Q}\;i}\right)}^{n}\;e^{-\;\frac{bqT}{Q}\;i\left(N-i-n\right)}$, for n=0,⋯,N

### 3.1 Model Parameters

Unless stated otherwise, parameters $b=0.4\;m^{3}\cdot h^{-1}$ (Pleil et al. 2021) and I=1 are considered in most of our results. For the quanta emission rate of an infector, we note that quanta generation rates can vary widely across many different orders of magnitude depending on a multitude of factors, including: pathogen of interest (Mikszewski et al. 2022; Chen et al. 2006), differences in physical activity (Jones et al. 2024; Buonanno, Morawska, et al. 2020; Aganovic et al. 2023), the stage of infection (Cheng et al. 2025; Ferretti et al. 2020; Sender et al. 2022; Wu et al. 2021; Euser et al. 2021), and individual heterogeneity such as age or gender (Jones, Biele, et al. 2021; Euser et al. 2021). For example, for SARS‐CoV‐2 the quanta emission rate across infectors has been described via a Lognormal distribution, LN(GM=0.0092,GSD=29), by Jones et al. (2024), spanning several orders of magnitude across individuals. In particular, this distribution illustrates the significant variability across individuals, with its arithmetic mean $\bar{q}=2.70\;\mathrm{quanta}\cdot h^{-1}$ representing the 95th percentile of the distribution. Indeed, significantly larger estimates have been obtained in the literature when studying particular large outbreaks during the pandemic, such as the Skagit county choir (Miller et al. 2021) and the Diamond princess cruise ship (Chen et al. 2021), which would correspond to top percentiles of the Lognormal distribution above.

Since our aim is to highlight the impact of parameter uncertainty in those scenarios where infection risk is non‐negligible, we focus here on scenarios where the infector is relatively highly infectious, corresponding to values near or above the mean value $\bar{q}=2.70\;\mathrm{quanta}\cdot h^{-1}$ estimated in Jones et al. (2024). In particular, values $q=1\;\mathrm{quanta}\cdot h^{-1}$ and $q=100\;\mathrm{quanta}\cdot h^{-1}$ are considered in some of our numerical results below. When aiming to illustrate the impact of uncertainty in q, we leverage instead the estimates provided by Chen et al. (2021) for the Diamond princess cruise ship; as such, the focus in this particular scenario in Section 3.2.2 is to consider uncertainty in q while focusing on a highly infectious individual, and our results in Figure 9 should be interpreted accordingly. In particular, to incorporate uncertainty while leveraging analytical results in Section 2, we use in Section 3.2.2 a Gamma distribution as an alternative to the Log‐normal distribution estimated by Chen et al. (2021), LN(184.64,2.1). Using the least squares method, we get

$$\begin{array}{ccc} q & ∼ & \text{Gamma}(\mathrm{shape}=2.39,\mathrm{rate}=0.012){\text{quanta h}}^{-1}; \end{array}$$

see Figure 3.

**FIGURE 3:**
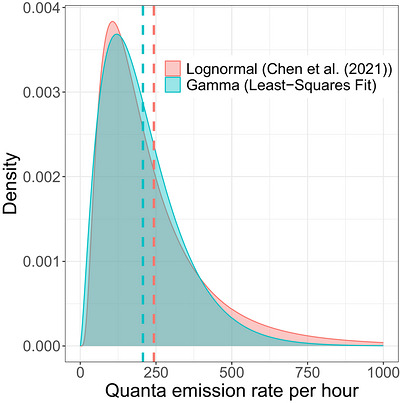
Gamma distribution for q with shape k=2.39 and rate λ=0.012, calibrated against the Lognormal distribution LN(GM =184.64, ASD =2.1) estimated by Chen et al. (2021) for the Diamond princess cruise COVID‐19 outbreak.

**FIGURE 4:**
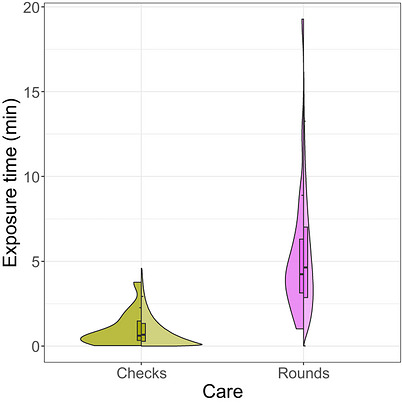
Split violin plots showing measured visit times (left side; King et al. 2021) with their best fitting Gamma distributions (right side) for each care type.

**FIGURE 5:**
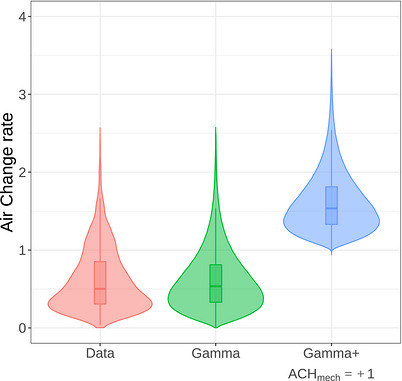
Violin plots for the natural ventilation data (ACH in red, from Edwards (2024)), and the calibrated gamma distribution $ACH_{nat}∼\text{Gamma}\left(2.52,4.10\right)$, with (blue, $ACH_{mech}=+1$) and without (green) mechanical ventilation.

**FIGURE 6:**
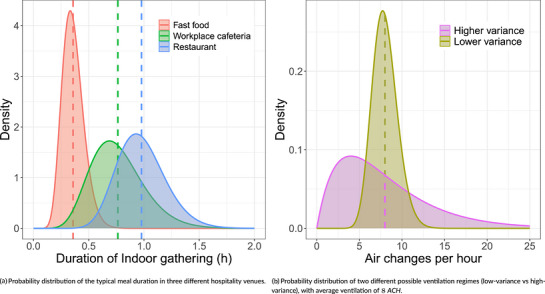
Probability distributions of the duration of the meal (T) and the ventilation rate (Q, plotted in ACH for room volume $V=300m^{3}$) in the hospitality scenario in Section 3.3 The vertical dashed lines represent the mean values of the distributions.

**FIGURE 7:**
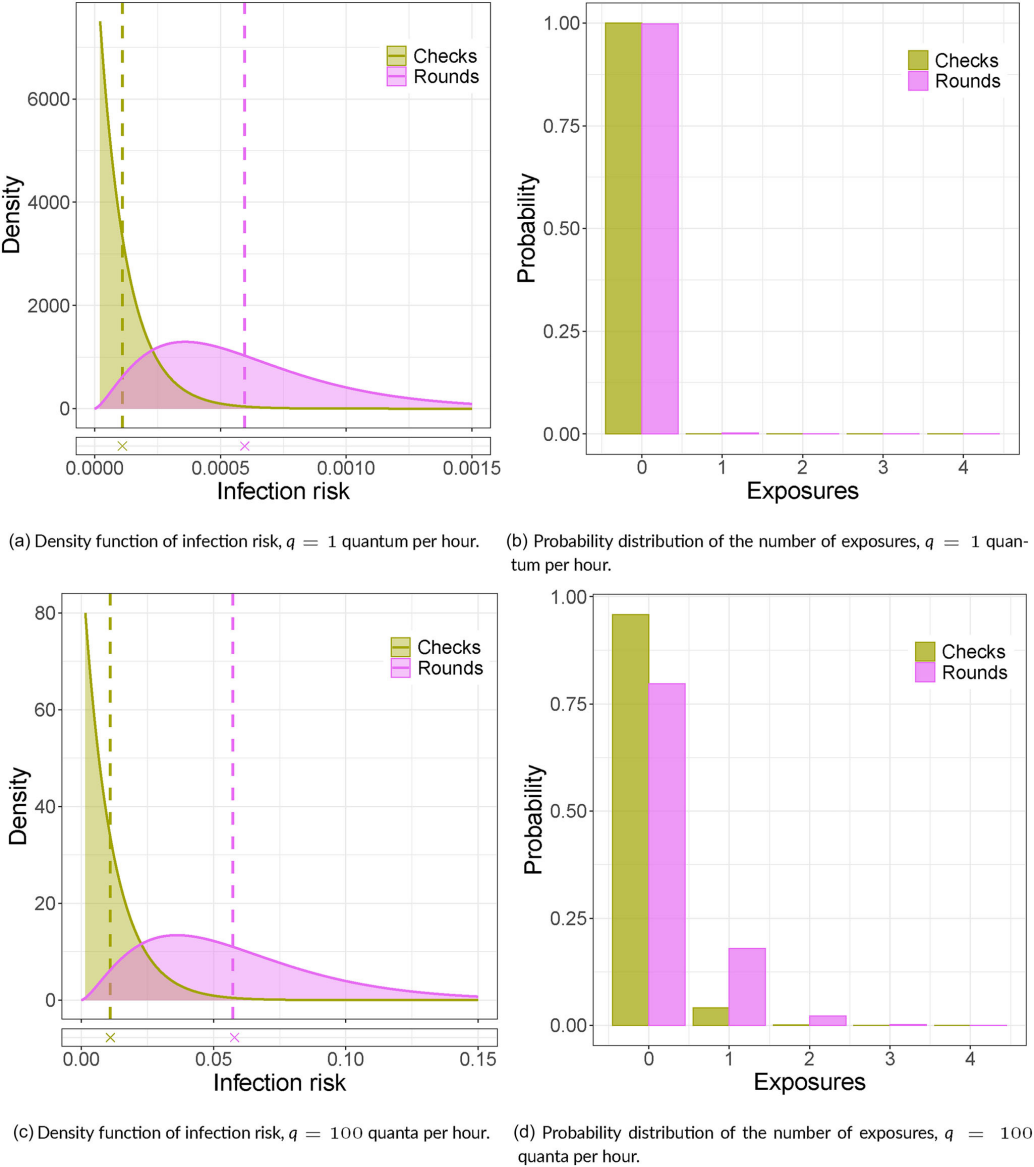
(a,c): Shaded density plots showing the distribution of the risk of infection with random visit times during each type of care (Equation (2) adapted to random T), for quanta emission rates of q=1 and $100\;\text{quanta}\cdot h^{-1}$. (b,d): Probability distributions of the number of exposures in the hospital ward scenario during each type of care calculated using Equation (4) adapted to random T (as seen in Table 1), for quanta emission rates of q=1 and $100\;\text{quanta}\cdot h^{-1}$.

**FIGURE 8:**
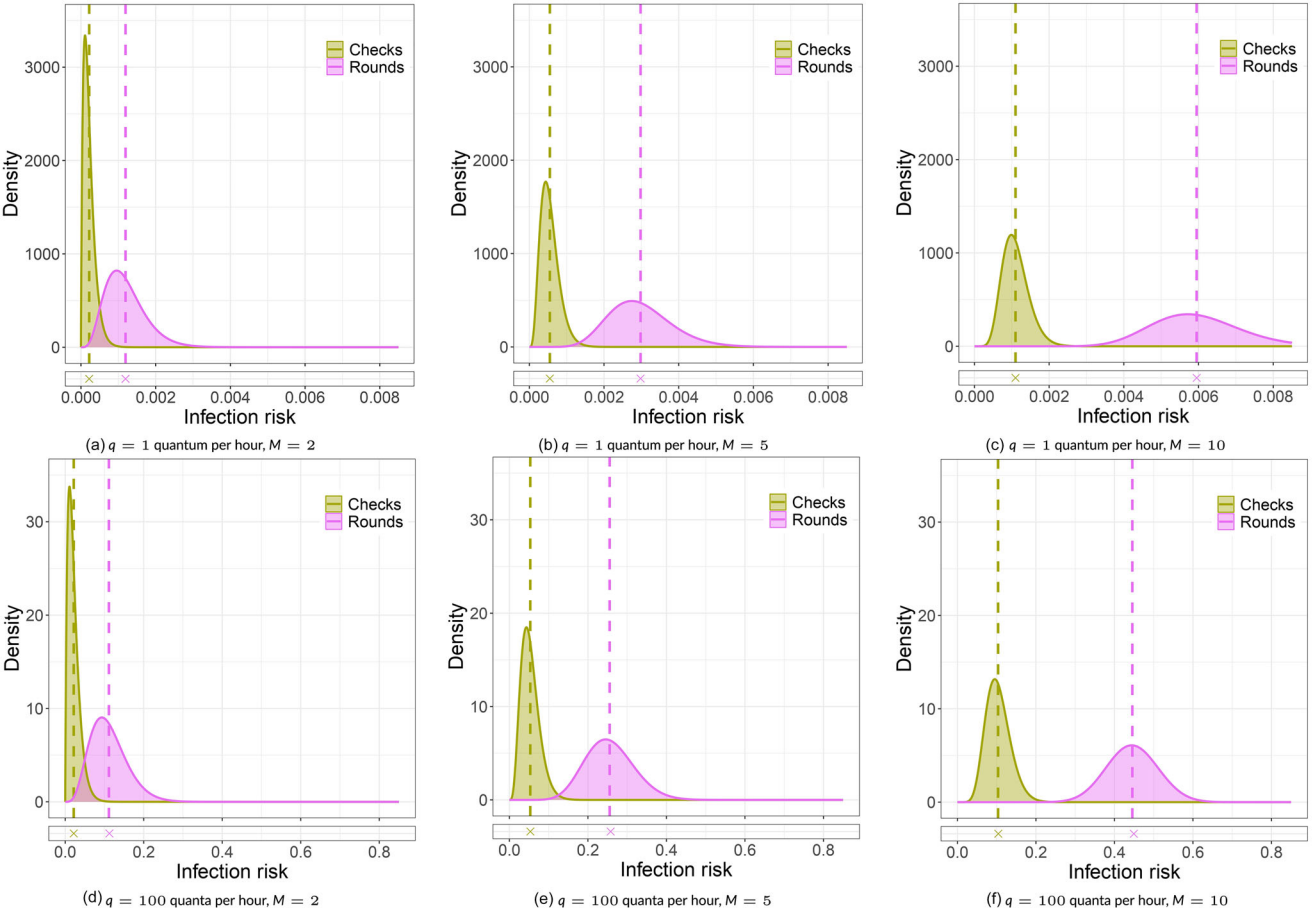
Density plots of the per‐capita infection risk probability (Equation (A15)), $f_{P_{infection}}\left(p\right)$, for a susceptible patient being visited sequentially M∈{2,5,10} times by an HCW, for either *Checks* (Yellow) or *Rounds* (Pink). Vertical dashed lines represent the mean infection risk $E[P_{infection}]$ (Equation (A16)). The crosses represent the classical Wells–Riley approach average infection risk ${\bar{P}}_{\mathrm{infe}\mathrm{ction}}^{\mathrm{WR}}=1-e^{-\frac{\mathrm{bq}}{Q}\bar{T}}$, with the mean visit time $\bar{T}$ as a constant value.

**FIGURE 9:**
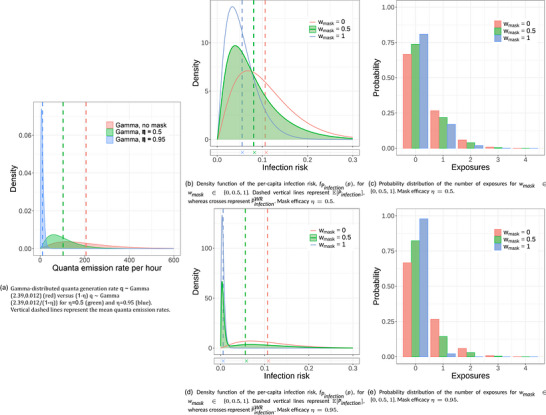
(a) Gamma‐distributed quanta generation rate q∼Gamma(2.39,0.012) (red) versus (1−η)q∼Gamma(2.39,0.012/(1−η)) for η=0.5 (green) and η=0.95 (blue). Vertical dashed lines represent the mean quanta emission rates. (b) and (d) Per‐capita infection risk density curves (Equation (5)) for mask efficacies η=0.5 and 0.95 and probabilities of the infector wearing the mask $w_{mask}\in \left\{0,0.5,1\right\}$. Vertical dashed lines represent mean infection risk, $E[P_{infection}]$ (Equation (6)), and the crosses represent the classical Wells–Riley estimate ${\bar{P}}_{infection}^{WR}=1-\exp\left(-\frac{IbT}{Q}{\bar{q}}_{eff}\right)$. (c) and (e) Probability distribution of the number of exposures (Equation (7)) for η=0.5 and 0.95 and probabilities of the infector wearing the mask $w_{mask}\in \left\{0,0.5,1\right\}$.

Finally, different parameter values (and distributions) for Q and T will be explored in Sections 3.2 and 3.3 when considering scenarios related to healthcare and hospitality. In particular, we consider in Section 3.2 a healthcare worker interacting with patients in a hospital ward, to illustrate the impact of uncertainty in ventilation, visit duration, and infectiousness. In Section 3.3, we consider infection risk during meals, to illustrate the impact of random prevalence as well as uncertainty in two parameter values simultaneously.

#### 3.1.1 Case Study 1: Healthcare Visits

In Section 3.2, we consider a similar scenario to that considered by Edwards, King, Noakes, et al. (2024) of a healthcare worker providing different types of care to patients in a hospital ward. In particular, and to illustrate how the duration of the indoor interaction may be uncertain, we leverage data from an observational study by King et al. (2021) which measured real and mock HCW visit times to a patient during various types of care (intravenous [IV] drip care, observational care, and doctor's rounds). We focus here on measurements corresponding to actual observational care for routine checks on patients (*Checks*) and doctor's rounds (*Rounds*), plotted in Figure 4. To account for this uncertainty when estimating infection risk while illustrating results in Section 2, we calibrate Gamma distributions to these two data sets by minimizing the negative log likelihood. This leads to distributions

$$\begin{array}{ccc} T_{\mathrm{Chec}\mathrm{ks}} & ∼ & \text{Gamma}(\mathrm{shape}=0.99,\mathrm{rate}=60)\;h,\\ T_{\mathrm{Roun}\mathrm{ds}} & ∼ & \text{Gamma}(\mathrm{shape}=2.51,\mathrm{rate}=28.1)\;h, \end{array}$$

which are plotted in Figure 4 against the corresponding data sets. In both cases, the distributions provide reasonable fits which allow one to capture the uncertainty around the corresponding mean visit duration and the unimodal shape of both data sets. We note that the duration of Checks $T_{\mathrm{Chec}\mathrm{ks}}$ has a narrower distribution (i.e., less uncertainty/variance) around $E[T_{\mathrm{Chec}\mathrm{ks}}]\approx 1\;\min$, whereas the duration of doctors' rounds is longer and more stochastic around its mean $E[T_{\mathrm{Roun}\mathrm{ds}}]\approx 5.36\;\min$.

Finally, uncertainty in ventilation rate is incorporated in the healthcare scenario by considering stochasticity in the natural ventilation rate as estimated by Edwards (2024), while accounting for the possibility of some constant mechanical ventilation being present in the ward. In particular, we use natural ventilation rate estimates from Edwards (2024), which accounted for weather factors such as wind direction, wind speed, and outside temperature. A Gamma distribution is calibrated against this data set by minimizing the negative log likelihood. This resulted in an air change rate of $ACH_{nat}∼\text{Gamma}\left(2.52,4.10\right)$ per hour, plotted in Figure 5 against the corresponding data set. This distribution is able to capture both the unimodal shape of the data set, with mode at around ACH=0.3, the possibility of very low ACHs near zero, and the tail representing ACHs up to 2.6. For a typical hospital ward volume of $V=98.35\;m^{3}$ (Edwards 2024), this leads to the ventilation rate

$$\begin{array}{c} Q_{\mathrm{nat}}∼\text{Gamma}(2.52,0.042)\;m^{3}\cdot h^{-1}. \end{array}$$

The possibility of adding some mechanical ventilation is depicted in Figure 5, which just shifts the Gamma distribution by a constant amount.

#### 3.1.2 Hospitality Parameters

In Section 3.3, we consider a similar scenario to the one considered by Edwards, King, Noakes, et al. (2024) to estimate infection risk during meals in different hospitality venues. In particular, Edwards, King, Noakes, et al. (2024) investigated the effects on infection risk from different durations of indoor gathering for different group sizes during the following lunch time scenarios: (i) a fast‐food restaurant, (ii) a workplace cafeteria, and (iii) a moderately priced standard restaurant. Edwards, King, Noakes, et al. (2024) leveraged observational data from Bell and Pliner (2003) and considered Erlang distributions for the typical duration of a meal in each venue,

$$\begin{array}{ccc} T_{Fast} & ∼ & \text{Erlang}(14,39.06)\;h,\\ T_{Cafeteria} & ∼ & \text{Erlang}(10,13.09)\;h,\\ T_{Restaurant} & ∼ & \text{Erlang}(20,20.45)\;h. \end{array}$$

Each of these distributions along with their mean values are reported in Figure 6a. Since Erlang distributions correspond to Gamma distributions with integer shape parameter, and to make our results comparable to those by Edwards, King, Noakes, et al. (2024), we consider the same distributions for our results in Section 3.3. We note that meal duration in the fast‐food restaurant is typically shorter, with less uncertainty, whereas durations increase in workplace cafeterias and restaurants, leading also to higher variability. We also note that the duration of these lunch times are longer and more varied than that of the visit durations in the healthcare setting in Figure 4.

Finally, and in order to illustrate in this scenario how uncertainty in two parameter values might simultaneously impact infection risk, we also consider in Section 3.3.1 two possible random ventilation rates corresponding to low variance and high variance around an average of 8ACH (Repace 2000; Casals Ventilation n.d.; Deutsches Institut für Normung 2019). In particular, for purely illustrative purposes, we consider (see Figure 6b)

$$\begin{array}{ccc} ACH_{highVar} & ∼ & \text{Gamma}(2,0.25),\\ ACH_{lowVar} & ∼ & \text{Gamma}(30,3.75), \end{array}$$

for our results in Figure 11. Importantly, both distributions have the same mean 8ACH, but different variances ($Var(ACH_{highVar})=32$ versus $Var(ACH_{lowVar})\approx 2$). Moreover, the ventilation regime with higher variance is more skewed towards smaller air change rates than the other ($Skew(ACH_{highVar})\approx 1.41$ versus $Skew(ACH_{lowVar})\approx 0.37$). A room volume of $300\;m^{3}$ is used in Section 3.3 as in Edwards, King, Noakes, et al. (2024), leading to ventilation rates

$$\begin{array}{c} Q_{\mathrm{high}\mathrm{Var}}∼\text{Gamma}(2,0.25/300){\;m}^{3}\cdot h^{-1},\\ Q_{\mathrm{lowV}\mathrm{ar}}∼\text{Gamma}(30,3.75/300)\;m^{3}\cdot h^{-1}. \end{array}$$

**FIGURE 10:**
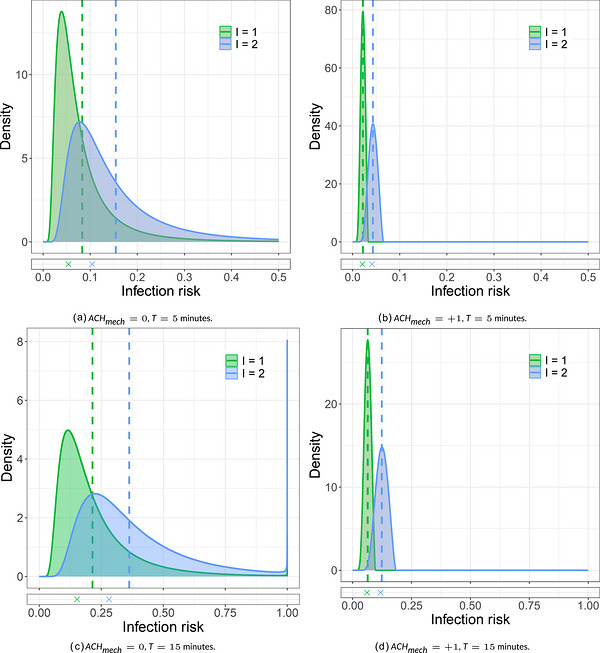
Probability density curves of the per‐capita infection risk, for natural ventilation only ($ACH_{mech}=0$) and added mechanical ventilation ($ACH_{mech}=1$), for exposure times T=5 and 15min, a quanta emission rate of $q=100\;{\text{quanta h}}^{-1}$, and for I=1 or I=2 infectors. Dashed vertical lines represent the distribution mean, $E[P_{infection}]$, and crosses represent the average value provided by classical Wells–Riley approach with average ventilation rate parameter, ${\bar{P}}_{\mathrm{infe}\mathrm{ction}}^{\mathrm{WR}}=1-\exp\left(-\mathrm{IbqT}/\left({\bar{Q}}_{\mathrm{nat}}+Q_{\mathrm{mech}}\right)\right)$.

**FIGURE 11:**
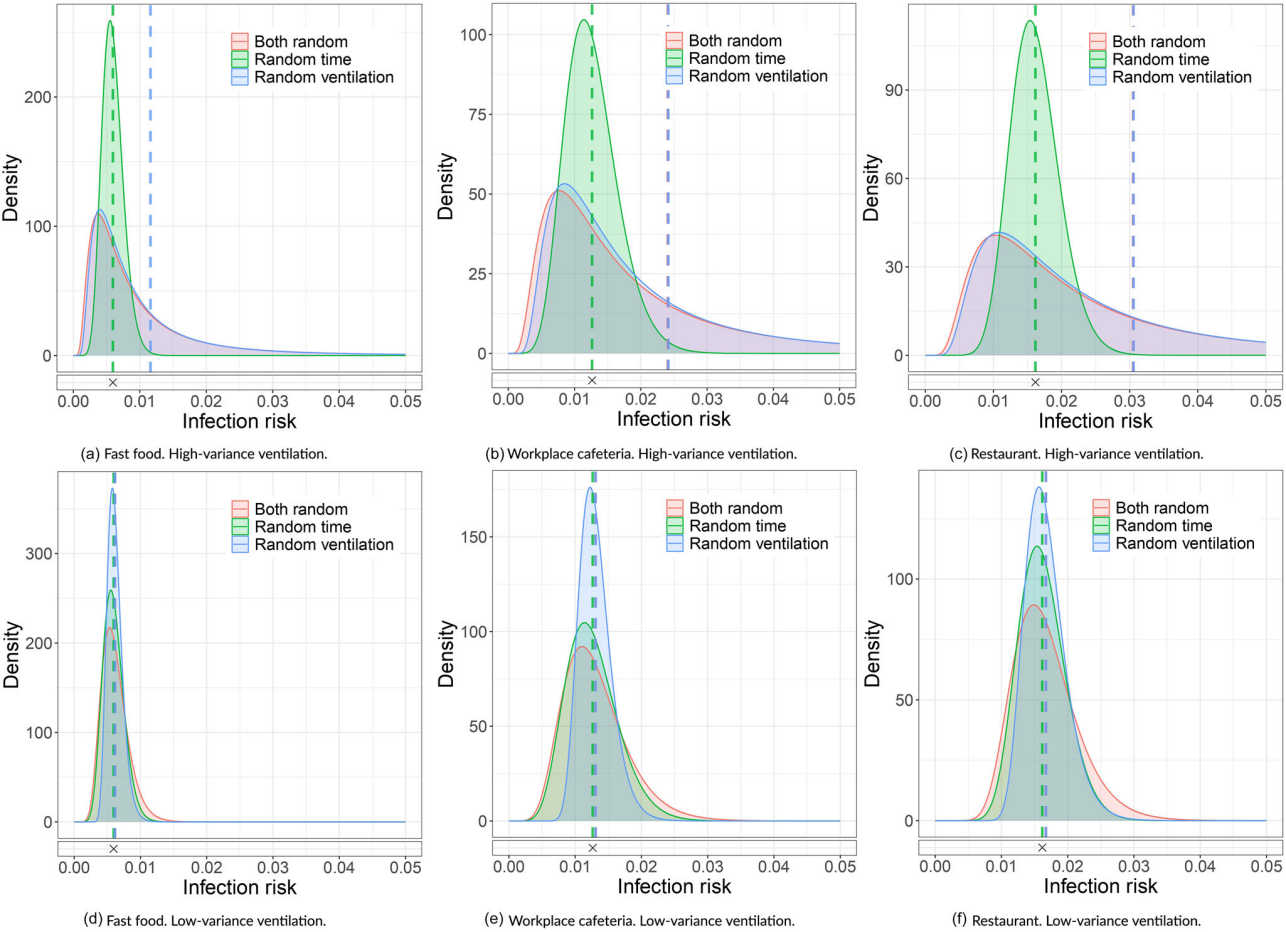
Density curves of the per‐capita infection risk probability $P_{infection}$ for different hospitality venues, given random T and Q (Equation (22)). For each scenario, we compare these density curves against the ones that one would obtain if one parameter is random and the other set to its mean value instead (Equation (2) and Equation (11)). Vertical dashed lines indicate density means (Equations (23), (3), and (12)). Crosses below the subplots indicate the classical Wells–Riley estimate ${\bar{P}}_{infection}^{WR}$ which uses average parameter values instead. Three hospitality venues with either high or low variance ventilation rates from Figure 6b.

### 3.2 Infection Risk During Healthcare

In this section, we analyze infection risk for S=4 patients in a hospital room being visited by an infectious healthcare worker while accounting for uncertainty in visit times, infectiousness, and ventilation rate, and by considering parametric regimes discussed in Section 3.1.1

#### 3.2.1 Uncertainty in Visit Times

The Gamma distributions for $T_{Checks}$ and $T_{Rounds}$ (see Section 3.1.1) provide a reasonably good representation of the empirical observations by King et al. (2021); see Figure 4, They also allow us to incorporate uncertainty in visit times while calculating infection risk via the extended Wells–Riley methodology proposed in Section 2. In particular, using these random visit times, we can use the analogous result to that in Equation (2) (just by swapping q with T, as well as their respective shape and rate parameters) to estimate the density function of the per‐capita infection risk for each patient in the room. These density functions $f_{P_{infection}}\left(p\right)$ of the per‐capita infection risk probability, for Checks and Rounds, are reported in Figure 7a,c, for q=1 and $100\;\mathrm{quanta}\cdot h^{-1}$, respectively, along with their expected values $E[P_{infection}]$ indicated by the vertical dashed lines.

First, we note that overall observational care (i.e., Checks) leads to lower infection risk compared to doctors' rounds (Rounds), which is to be expected given the shorter duration during Checks. Less visit duration uncertainty during Checks (i.e., a narrower Gamma distribution compared to that of Rounds) leads to less uncertainty in the corresponding infection risk probability (i.e., narrower density function $f_{P_{infection}}\left(p\right)$ for Checks compared to Rounds). On the other hand, more uncertainty in the duration of doctors' rounds leads to higher uncertainty in the infection risk for susceptible patients in the room. This leads to a density function of infection risk around the mean values $E[P_{infection}]\approx 0.0006$ (for $q=1\;\mathrm{quanta}\cdot h^{-1}$) and $E[P_{infection}]\approx 0.057$ (for $q=100\;\mathrm{quanta}\cdot h^{-1}$) but showing larger variability for Rounds compared to Checks. For example, in the case of $q=100\;\mathrm{quanta}\;h^{-1}$, infection risk might be significantly higher (up to 0.15) for longer Rounds episodes or smaller (down to almost 0) for shorter ones, with a mode infection risk probability of $Mode(P_{infection})\approx 0.04$. We note that the shape of the per‐capita infection risk density functions in Figure 7 is preserved across q=1 and $100\;\mathrm{quanta}\cdot h^{-1}$ for each type of care. In fact, for a particular type of care, these densities scale linearly (by around $10^{2})$ between $q=1\;\mathrm{quanta}\cdot h^{-1}$ and $q=100\;\mathrm{quanta}\cdot h^{-1}$, since for small enough infection risk the Wells–Riley model equation becomes approximately linear on q. However, this is not the case for the n‐exposure distributions plotted in Figure 7b,d, for n=0,1,2,3,4 for a room with S=4 susceptible patients. For $q=1\;\mathrm{quanta}\cdot h^{-1}$, the per‐capita infection risk is small enough so that we see no notable differences in the distribution of exposures between Rounds and Checks. On the other hand, for $q=100\;\mathrm{quanta}\cdot h^{-1}$ longer visit times for Rounds lead to a rightward‐shift in the corresponding distribution, where the probability of at least 1 exposure is non‐negligible.

**FIGURE A1:**
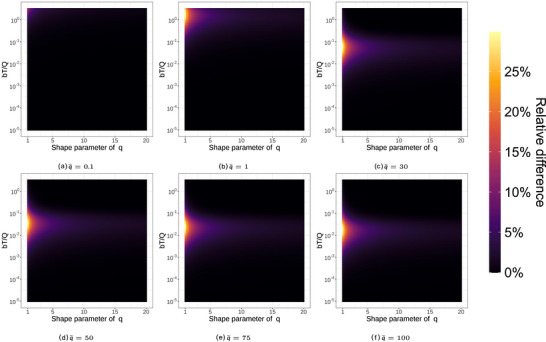
Heatmaps for the relative difference between ${\bar{P}}_{\mathrm{infection}}^{\mathrm{WR}}$ (classical Wells–Riley approach with constant, average quanta generation rate $\bar{q}=k/\lambda$), and the analytical mean infection risk $E[P_{infection}]$ in Equation (3), versus k, for $\bar{q}=0.1,1,30,50,75,100\;{\text{quanta h}}^{-1}$.

It is interesting to compare our results, which allow us to better quantify uncertainty in infection risk, with the classical Wells–Riley approach where the visit time is considered to be known and fixed. In particular, we can compute the per‐capita infection risk probability ${\bar{P}}_{infection}^{WR}=1-e^{-\frac{Ibq}{Q}\bar{T}}$ while setting the visit time as the mean visit time for each type of care in Figure 4; that is by setting $\bar{T}=E[T_{\mathrm{Chec}\mathrm{ks}}]\approx 1\;\min$ for Checks and $\bar{T}=E[T_{\mathrm{Roun}\mathrm{ds}}]\approx 5.36\;\min$ for Rounds. This infection risk probability computed via the classical Wells–Riley approach is reported as a colored cross at the bottom of the density plots in Figure 7. In these scenarios, the classical Wells–Riley approach (using the mean visit duration) gives a very similar infection risk probability ${\bar{P}}_{infection}^{WR}$ to the expected value $E[P_{infection}]$ (dashed vertical lines) calculated via our methodology (which summarizes the uncertainty around T in an average infection risk value). For $q=1\;\mathrm{quanta}\cdot h^{-1}$ (Figure 7a), we have bq/Q=2/300, for which we know from Figure 1 (such that $\bar{q}$ is replaced by $\bar{T}$ and bT/Q is replaced by bq/Q) overestimations do not occur in the region of ${\bar{T}}_{\mathrm{Roun}\mathrm{ds}}\approx 10^{-1}\;h$, ${\bar{T}}_{\mathrm{Chec}\mathrm{ks}}\approx 10^{-2}\;h$ and $bq/Q\approx 10^{-2}$. However, there are other situations where using the classical Wells–Riley approach can lead to a more significant overestimation of infection risk as described in Subsection 2.1.1. By looking at Figure A1 in Appendix A5.1, we can see that for bq/Q being on the order of $10^{-2}$, we would need $\bar{T}=30\;h$ before any noticeable relative difference can be seen. Similarly, for $q=100\;\mathrm{quanta}\;h^{-1}$ we have bq/Q=2/3, for which we would require $\bar{T}=1\;h$ before overestimations start to occur.

**FIGURE 12:**
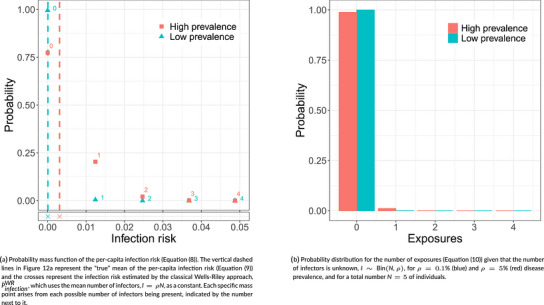
Infection risk in the workplace cafeteria for $\bar{T}=45\;\min$ and 8ACH, with a random number of infectors I∼Bin(N,ρ), disease prevalence ρ=0.1% (low, blue) and ρ=5% (high, red), and N=5 individuals in total.

A particular feature of our approach (considering Gamma‐distributed parameters) is that one can, for example, consider the impact that sequential visits have on infection risk. In particular, since the sum of independent Gamma distributions is a Gamma distribution, one can consider sequential visits from HCWs to a given room. For example, for M>1 sequential Checks (e.g., during a day), assuming that each observational care visit to the room is carried out independently with duration $T_{\mathrm{Chec}\mathrm{ks}}∼\text{Gamma}(0.99,60)h$, the total time spent by the HCW in the room is $T_{\mathrm{Tota}\mathrm{lChe}\mathrm{cks}}∼\text{Gamma}(0.99M,60)h$. Thus, due to the steady‐state assumption in the Wells–Riley framework, the overall infection risk from M sequential visits (each of duration $T_{Checks}$) is equal to the infection risk from a single visit with duration $T_{TotalChecks}$, which can be analyzed via our methodology in Section 2.1, since it is Gamma‐distributed; see Appendix A4 for further details.

In Figure 8, we plot the density function of the infection risk probability for a susceptible patient from M∈{2,5,10} sequential HCW visits (either Checks or Rounds), for rates of $q=1{\;\text{quanta}\cdot h}^{-1}$ and $q=100\;{\text{quanta}\cdot h}^{-1}$. As expected, an increasing number of visits from an infector to the room significantly increases infection risk in both care‐type scenarios. It is however interesting to see how an increasing number of visits compounds uncertainty in each visit duration (leading to higher uncertainty in infection risk), where even a narrow Gamma distribution for $T_{Checks}$ leading to low risk uncertainty in Figure 7a,c now leads to notably higher uncertainty in infection risk for M=2,5, and 10 sequential Checks in Figure 8. This highlights the importance of considering uncertainty in parameter values when estimating infection risk in these types of situations. As the number of sequential visits increases, the increase in infection probability between the cases $q=1{\;\text{quanta}\cdot h}^{-1}$ and $q=100{\;\text{quanta}\cdot h}^{-1}$ becomes nonlinear; we still observe uncertainty around infection risk but the relative magnitude of this uncertainty is no longer the same for the two emission rates; this is particularly true for Rounds in Figure 8c,f.

#### 3.2.2 Uncertainty in Infectiousness

Here, we analyze the impact of uncertainty in quanta generation rate by considering $q∼\text{Gamma}(2.39,0.012)\text{quanta}\cdot h^{-1}$ as in Figure 3, while illustrating results in Section 2.1.1 to incorporate the possibility of the infector wearing a mask with probability $w_{mask}$, which reduces the corresponding generation rate by a factor of η. As a result, randomness in infectiousness may be due to uncertainty in the quanta generation rate estimate (Figure 3) or due to human behavior. In Figure 9, we explore two different values of mask efficacies of 50% (η=0.5) and 95% (η=0.95), to explore a wide range of values which can be affected by mask type and quality of fit (Davies et al. 2013; Wilson 2020; Bazant and Bush 2021; Rothamer et al. 2021; Huang et al. 2022). The impact of η on the corresponding Gamma‐distributed quanta generation rate is plotted in Figure 9a. For these two values of η, we plot the infection risk densities in Figure 9b,d for probabilities $w_{mask}\in \left\{0,0.5,1\right\}$ of the infector wearing the mask, as well as the probability distribution of the number of exposures in Figure 9c,e. We consider an interaction of T=5min in all these scenarios. When focusing on the density curve of the per‐capita infection risk in Figure 9b, one can note that $w_{mask}=1$ (infector wears mask, blue curve) generally leads to lower risk compared to $w_{mask}=0$ (infector does not wear mask, red curve), as one would expect. Still, high uncertainty in these generation rates (variance of the corresponding Gamma distributions in Figure 9a) means that these red and blue density functions overlap. Thus, for $w_{mask}=1$, one could still get relatively high values of $P_{infection}$ (i.e., per‐capita infection risk above 0.15 corresponding to the right tail of the blue density curve) while for $w_{mask}=0$ one could still get low infection risk (i.e., per‐capita infection risk close to 0 in the left part of the red density curve), with non‐negligible probabilities. Setting $w_{mask}=0.5$ (the infector has an equal chance of wearing or not wearing the mask) means that the density curve of the probability of infection shares characteristics with both of the underlying densities (for $w_{mask}=0$ and 1), which is particularly interesting in the case η=0.95 (Figure 9d). In this case, the high mask efficacy η reduces the effective quanta emission by enough (blue vs. red in Figure 9a) that the underlying distributions have little overlap. This highlights how stochasticity in human behavior ($w_{mask}$) can have a high impact on infection risk in this type of situation. In particular, the large value of η=0.95 leads to bi‐modality in the resulting (green) density curve for the per‐capita infection risk in Figure 9d, representing two significantly different per‐capita infection risk regions depending on the individual wearing or not wearing the mask. In this situation, it is clear that the mean infection risk (dashed vertical green line in Figure 9d) or the single estimate (green cross) provided by the classical Wells–Riley approach with average quanta generation rate ${\overset{-}{q}}_{\mathrm{eff}}$ are not good representations of the true infection risk profile (given by the whole green density curve in Figure 9d). In these type of high‐stochasticity high‐impact scenarios, our results highlight the importance of considering this uncertainty when predicting risk, rather than relying on average/fixed parameter values or just focusing on average per‐capita infection risk estimates.

Finally, the n‐exposure distributions have been plotted in Figure 9c,e. The reduction of approximately 2% (8.3% to 5.9%) in the mean per‐capita infection risk (for $w_{mask}=0.5$) between η=0.5 to η=0.95 leads to a slightly leftwards shift in the n‐exposure distribution. The probability of at least one exposure occurring increases notably for smaller $w_{mask}$ and η. For a larger value of η (0.95 compared to 0.5), the impact of mask‐wearing becomes larger; this means that the n‐exposure distributions are more different for different values of $w_{mask}$ when η=0.95 compared to 0.5. That is, for decreasing efficacy values η→0 (representing decreasing fit or mask quality), the differences between different values of $w_{mask}$ would become less significant.

#### 3.2.3 Uncertainty in Ventilation Rate

In this section, we investigate the effects of random ventilation by considering the distributions in Figure 5. In particular, we plot in Figure 10 the per‐capita infection risk density curves under only natural ventilation ($Q_{mech}=0$) or the presence of some mechanical ventilation ($ACH_{mech}=+1$), while considering two visit durations (T=5min vs. T=15min) and I∈{1,2}. We consider the quanta generation rate $q=100{\;\text{quanta h}}^{-1}$ here to represent high infectivity; a shift on infection risk similar to that observed in Figure 7 would be expected if one considered $q=1\;\mathrm{quanta}\;h^{-1}$ instead.

As reported by Edwards, King, López‐García, et al. (2024), reliance on natural ventilation alone can lead to high‐risk situations during moments (or days) where the particular environmental conditions lead to significantly low ventilation rates in a given room, represented by values of $Q_{nat}$ close to 0 in the corresponding Gamma distribution in Figure 5. Indeed, these values lead to long “high‐risk” tails of the per‐capita infection risk density curves, close to 40% in Figure 10a and close to 100% in Figure 10c, especially if I=2 or T=15min. It is also interesting to note how the same level of uncertainty in Q (in terms of the same variance for the Gamma distributions in Figure 5) between the different scenarios considered (natural ventilation only in Figure 10a,c, vs. additional mechanical ventilation in Figure 10b,d) can lead to substantially different levels of uncertainty in the per‐capita infection risk. In particular, this level of uncertainty in Q has a more significant impact when only natural ventilation is considered (which leads to some days, as described, with very low ventilation rates near 0). Once some mechanical ventilation ($ACH_{mech}=+1$) is added, so that the distribution of Q is shifted in Figure 5, uncertainty in the per‐capita probability of infection significantly decreases, consistent with findings in Edwards, King, López‐García, et al. (2024). This highlights the impact that having mechanical ventilation in the room, even a small amount, may have on infection risk when the chances of having very low natural ventilation rates near 0 are non‐negligible in that particular environment.

When considering uncertainty in Q, the classical Wells–Riley approach (which consists of computing ${\bar{P}}_{infection}^{WR}=1-e^{-\frac{IbqT}{\bar{Q}}}$, with $\bar{Q}={\bar{Q}}_{nat}+Q_{mech}$ being the average total ventilation rate) typically leads to an underestimation of risk in Figure 10, consistent with our results in Figure 2. This is due to the fact that using the average ventilation rate partly neglects high‐risk situations where, for example, reliance on natural ventilation leads to situations with very low ventilation rates on particular days, as discussed above; see also Appendix A5.2 for further details. Overall, our approach, where ventilation rates and infection risk are represented as random variables, allows one to capture the probability (and impact) of these events when estimating infection risk in the presence of uncertainty.

### 3.3 Infection Risk During Meals

Here, we consider a similar scenario to the one considered by Edwards, King, Noakes, et al. (2024) to estimate infection risk during meals in different hospitality venues, and while accounting for uncertainty in two parameter values simultaneously, or in disease prevalence.

#### 3.3.1 Uncertainty in Meal Duration and Ventilation Rate

We investigate here the impact of considering simultaneous uncertainty in T and Q in this scenario, while leveraging the Gamma distributions described in Section 3.1.2. Once again, we set $q=100{\;\text{quanta h}}^{-1}$ here for illustrative purposes, corresponding to a highly infectious individual in the venue. We use Equations (22) and (23) (with q substituted by T) to calculate the density curves and expected values of $P_{infection}$ given our different scenarios and ventilation rates. These are all plotted in Figure 11. Within each subplot, we consider the situation where only one parameter is considered to be random (either T or Q), while the other is set to the mean value of its corresponding Gamma distribution, or where both parameters are simultaneously random.

Overall, infection risk is lower in fast‐food venues compared to workplace cafeterias or restaurants, due to shorter lunch durations. More variability in lunch duration in these latter venues also leads to higher variability in infection risk. Still, stochasticity in ventilation rate “dominates” (i.e. against randomness of T) the stochasticity in infection risk when the ventilation rate distribution has higher variance and a higher positive skewness (Figure 11a–c), which can be noticed by the red and blue density curves almost overlapping in these scenarios. Once again, the distribution of the ventilation rate in the high‐variance scenario in Figure 6b leads to low ventilation rates with high probability, even though the mean ventilation rate is still 8ACH. This directly contributes to the long right tail of the density function of $P_{infection}$ in Figure 11a–c (red and blue curves), which is missing when only randomness in T is considered (green curve). We refer the reader to Appendix A6 where we investigate further the reasons for one parameter to dominate the shape of the density function of $P_{infection}$ over the other, when both parameters are random. On the other hand, the low‐variance ventilation rate scenario in Figure 6b leads to a very low probability of observing a ventilation rate below 5ACH at any given time. In this case, randomness in T becomes more relevant when estimating the density function of $P_{infection}$. This can be seen in Figure 11d–f, where the red density curve (both parameters considered to be random) does not closely mimic any of the other two densities (blue and green). Interestingly, the red density curve always has larger variance compared to the green and blue ones. This highlights the compounding effect that having uncertainty in two parameter values simultaneously has on the uncertainty in the per‐capita infection risk. That is, for example, if both T and Q are random, there will be scenarios where a particularly long lunch duration combined with a particularly low ventilation rate (sampled from the corresponding parameter distributions) could lead to increased infection risk, leading to the relatively longer right tail of the red density curve in Figure 11d–f.

We can also compare our predictions with the per‐capita infection risk estimated via the classical Wells–Riley approach when both parameters are set to be constant equal to their mean values, represented by the crosses in Figure 11. The most striking difference is that observed between these values (crosses, representing the classical Wells–Riley approach ${\bar{P}}_{infection}^{WR}$) and the dashed vertical red and blue lines (which are on top of each other, representing analytical mean infection risks $E[P_{infection}]$) in Figure 11a–c, where we observe underestimations by the classical approach of approximately 50% relative to the analytical means. This highlights how the classical (average) Wells–Riley approach would clearly underestimate infection risk if uncertainty in Q is not properly accounted for. On the other hand, the fact that the red and blue lines overlap, whereas the green vertical dashed line is significantly below and closer to the cross, means that in this type of scenario considering uncertainty in Q is enough to capture most of the real uncertainty in $P_{infection}$, whereas the uncertainty in T becomes less important here and could be neglected.

#### 3.3.2 Uncertainty in Number of Infectors: Considering Disease Prevalence

It is typical when assessing infection risk to assume that an infector is present. However, the probability of an infector being present is one of the main factors influencing infection risk for susceptible individuals in reality. This can be incorporated, as discussed in Section 2.1.2, via a probability distribution for I. If we consider the population‐level probability of being infectious to be ρ, and assuming independence, one can consider I∼Binomial(N,ρ) where N is the number of individuals involved in the indoor interaction. Looking at the trends for the percentage of positive COVID‐19 tests during 2022 (Office for National Statistics 2023), we can see that they typically ranged between 1% and 8%. Thus, for illustrative purposes, we consider two potential values of ρ∈{0.001,0.05} to represent low and high prevalence scenarios, respectively. We define two variables for the number of infectors

$$\begin{array}{ccc} I_{\mathrm{low}} & ∼ & \text{Binomial}(5,0.001),\\ I_{\mathrm{high}} & ∼ & \text{Binomial}(5,0.05). \end{array}$$

As an example, we will consider the workplace cafeteria scenario, fixing $\bar{T}=10/13.09\;h\approx 45\;\min$ to be the corresponding mean lunch duration and a ventilation of 8ACH. Equations (8) to (10) are used to calculate the probability distribution of the per‐capita infection risk as well as the probability of observing n exposures. The per‐capita infection risk distributions and their means have been plotted in Figure 12a, along with the classical Wells–Riley probability of infection calculated by fixing $\bar{I}=N\rho$ as the mean number of infectors. We note that since I is a discrete random variable, the per‐capita infection risk becomes now a discrete random variable as well, which is why we plot a probability mass function in Figure 12a instead of a density curve.

When we have a prevalence of ρ=0.1%, it is highly unlikely for anyone present at the lunch to be infectious, leading to the per‐capita infection probability being 0 with a likelihood close to 100%. On the other hand, when the prevalence is increased to ρ=5%, the probability of at least one infectious individual being present becomes non‐negligible (P(I>0)=22.6%), which explains why the red probability mass function in Figure 12a attributes non‐negligible likelihood to positive per‐capita infection risks. Still, the magnitude of the per‐capita infection risk remains low in all cases in Figure 12a (less than 5%), which is explained by the high ventilation rate considered. For both prevalence rates, the Wells–Riley approach seems to capture the density mean well, possibly because risks are low in this scenario.

In Figure 12b, we have plotted the probability distribution of the number of exposures within the group. The high probability of observing 0 exposures, even under high prevalence, highlights the importance of taking into account the probability of an infector being present when carrying out infection risk assessment (rather than assuming that an infector is in the room). This is directly related to the idea of implementing mitigations which may avoid an infector being present in the first place (e.g., avoiding contact if symptomatic), above other additional mitigations which might be considered.

## 4 Discussion

In this study, we revisited the classical Wells–Riley model by introducing a stochastic framework that analytically accounts for uncertainty in key parameters such as the quanta emission rate, ventilation rate, exposure time, and number of infectors. Unlike existing approaches in the literature which are typically based on numerical or Monte Carlo simulation methods, we propose a probabilistic, mathematical framework which extends the work by Nicas (1996) and Edwards, King, Noakes, et al. (2024). In particular, by treating these parameters as random variables, we derived probability distributions for both the per‐capita infection risk and the number of exposures during indoor interactions. When compared to a classical Wells–Riley model with average parameter values, our probabilistic approach allows for a more nuanced and realistic assessment of airborne infection risk, particularly in heterogeneous populations and variable environmental conditions. It is worth noting that there exist dose–response models which use an exponential‐Poisson relationship to infection probability similar to that of the Wells–Riley model, and authors have previously incorporated uncertainty into the dose (Sze To and Chao 2010; Nicas 1996). In particular, Nicas (1996) investigates Beta‐distributed and Gamma‐distributed doses, and derives infection probability distributions. Here, we instead consider uncertainty in each of the parameters which can impact the dose within the Wells–Riley framework.

Our findings demonstrate that the classical Wells–Riley model under average parameter values can lead to significant inaccuracies when estimating infection risk. A particularly interesting result is that generated in Section 2.1.1 which leverages Jensen's inequality: The per‐capita infection risk will be overestimated if one implements the classical Wells–Riley methodology under average parameter values for (b,q,T), compared to computing the “true” average per‐capita infection risk which incorporates the whole probability distribution of the corresponding parameter. On the other hand, considering an average ventilation rate typically leads to underestimating the true per‐capita infection risk. This is particularly significant in scenarios where very poor ventilation can occur with positive probability; in these situations, considering an average ventilation rate neglects the possibility of these high‐risk events happening. These insights underscore the importance of incorporating parameter uncertainty into infection risk assessments, especially in settings where precise measurements are unavailable or impractical. It also highlights the importance of incorporating low‐probability high‐impact events (e.g., scenarios with very low ventilation, the presence of a highly infectious individual or the possibility of this infector wearing a mask), which can be done via the analytical framework here proposed.

Overall, large uncertainty in parameter values leads to large uncertainty in the per‐capita infection risk estimates. The nature of this resulting uncertainty can significantly change across scenarios though. For example, we illustrated in Figure 9d how, in some scenarios, bi‐modality might arise where particular stochastic conditions or random human behavior might lead to very different infection risk profiles depending on a particular random event (in this figure, the infector wearing or not a mask). In these situations, it is clear how considering either average parameter values or average infection risk estimates would not be representative or informative of the real infection risk profile outlined by the green density in Figure 9d. This has direct implications in terms of risk mitigation design, where, for example, here one would focus on decreasing risk in those scenarios corresponding to the second larger mode in Figure 9d (e.g., by increasing $w_{mask}$) rather than trying to shift the whole probability density curve to the left via alternative mitigations.

Our results also highlight how, when considering two parameters being simultaneously random, uncertainty in one of these parameters can in some scenarios dominate the uncertainty in the corresponding per‐capita infection risk, as explored in Figure 11. This has direct implications in terms of both incorporating this uncertainty into the risk estimates and considerations around data collection. That is, if uncertainty around the ventilation rate is expected to have the largest impact on the infection risk estimates, one would need to incorporate this uncertainty into the corresponding mathematical framework (as described in Section 2), as well as rely on better measurements for this particular parameter. In these instances, variability in other parameters may be neglected if this variability is expected to be smaller or have a smaller impact on infection risk. Our methodology allows one to explore this in detail when carrying out infection risk assessment in different settings.

Proposing an analytical framework as the one in Section 2 allows one to carry out extensive sensitivity analysis under different parametric regimes, as well as to leverage analytical properties as we did with Jensen's inequality in Section 2.1.1. Moreover, this analytical, probabilistic framework has the potential to be extended to additional scenarios (e.g., alternative parametric distributions) or when relaxing particular assumptions (e.g., steady‐state pathogen concentration in the air). While some of the closed formulas obtained in Section 2.1, such as Equations (2)–(11) and (15), can be readily applied, other results in this work depend on integral functions such as the modified Bessel or the Tricomi function. Still, these are well‐known functions in the literature for which analytical properties and numerical implementations are readily available, and we leveraged these in some of our results in Section 3. Thus, our analytical results could be easily embedded into existing QMRA frameworks, allowing one to quickly test uncertainty in various scenarios without a major need in computing power. These analytical results could also be used directly in Bayesian frameworks when estimating model parameters from observations, since some of our results would arise when computing likelihood functions in these scenarios.

Despite the strengths described above, several limitations should be acknowledged. First, our approach is derived from the classical Wells–Riley model, which uses the steady‐state assumption for the concentration of infectious pathogen in the air. For short exposure interactions combined with low ventilation, the steady‐state assumption can overestimate infection risk, as shown in Edwards et al. (2023). This limitation has been addressed by Gammaitoni and Nucci (1997), who extended the Wells–Riley model to include transient concentration dynamics (exponential growth or decay) (see also Edwards et al. 2023; López‐García et al. 2019). Our approach could be strengthened by incorporating parameter uncertainty into that transient model.

Second, we have considered at most two (independent) parameters being simultaneously random when estimating infection risk. In reality, uncertainty might be present in more than two parameters, or some parameters may exhibit correlations (e.g., between infectiousness and behavior, or between ventilation patterns and occupancy). Although the methodology could in principle be extended to more than two random parameters, or pairs of correlated parameters (e.g., via joint distributions), the resulting analytical expressions may soon become intractable. In these situations, numerical or Monte Carlo simulation approaches as those existing in the literature (Bate et al. 2024; Henriques et al. 2025; Iddon et al. 2022; Jones, Sharpe, et al. 2021; Jones et al. 2025; Edwards et al. 2026) would be preferred.

Third, we have considered Gamma‐distributed parameters in Section 2 due to their mathematical tractability and versatility, going beyond Exponential or Erlang‐distributed ones as considered by Edwards, King, Noakes, et al. (2024). The flexibility of Gamma distributions to represent different data sets has been shown in Section 3, where bi‐modal parameter regimes or sequential indoor interactions could be accounted for by considering different combinations of Gamma distributions. However, there might be situations where different distributions might provide a better fit to data for a particular parameter, in which case our results would need to be extended, if possible. For example, quanta emission rates have been modeled as log‐normal in the literature with variation across multiple orders of magnitude (Jones et al. 2024). Future work might be able to partially address this limitation. For example, the analytical mean infection risk in Equation (3) can be written in terms of the Laplace transform of the random parameter. We recall that for a random variable X with density $f_{X}\left(\cdot \right)$, the Laplace–Stieltjes transform is defined as $L_{f_{X}}\left(s\right)=\int_{0}^{\infty }e^{-sx}f_{X}\left(x\right)\;dx$. Then, the mean infection risk $E[P_{\mathrm{infection}}]$ for a generally distributed quanta emission rate q∼G(·) can be computed as

$$\begin{array}{ccc} E[P_{\mathrm{infection}}] & = & 1-\int_{0}^{\infty }e^{-\frac{IbqT}{Q}}f_{G}\left(q\right)\;dq=1-L_{f_{G}}\;\left(\frac{IbT}{Q}\right), \end{array}$$

which could be applied to any distribution with a well‐defined Laplace–Stieltjes transform.

Future work could extend this framework by integrating more detailed behavioral and environmental data or exploring nonparametric or data‐driven distributions. Moreover, incorporating real‐time monitoring of indoor air quality and occupancy could enable dynamic risk assessments and inform adaptive mitigation strategies. Ultimately, our stochastic extension of the Wells–Riley model provides a valuable analytical tool for robust and precautionary infection risk estimation in diverse indoor settings under environmental and behavioral uncertainty, and population heterogeneity. These insights into the impact of parameter uncertainty in infection risk assessment have important implications for pandemic preparedness and resilience, particularly given the potential for emergence of novel airborne pathogens through reassortment and other evolutionary mechanisms. Segmented RNA viruses pose particular concern, as reassortment between co‐infecting strains can generate progeny with altered transmission characteristics, exemplified by influenza pandemic strains (Neumann et al. 2009; Taubenberger and Kash 2010). Similarly, Bunyaviruses with tripartite segmented genomes demonstrate significant reassortment potential that could alter virulence and transmissibility (Briese et al. 2013; Rezelj et al. 2019). While most are vector‐borne, some Bunyaviruses show person‐to‐person transmission capabilities (e.g., Andes Hantavirus via aerosols (Martinez et al. 2005) and SFTS virus through contact transmission (Gai et al. 2012)) suggesting biological potential for enhanced airborne transmission in reassortant strains. Our stochastic framework's ability to account for uncertainty in human behavior and environmental conditions provides essential tools for rapidly assessing transmission risks from such emerging pathogens, where precise parameter estimates may be unavailable during initial outbreak responses.

## Conflicts of Interest

The authors declare no conflicts of interest.

## Figures and Tables

**FIGURE A2:**
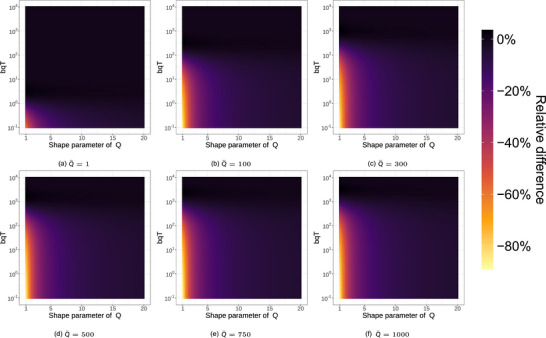
Heatmaps for the relative difference between ${\bar{P}}_{\mathrm{infection}}^{\mathrm{WR}}$ (classical Wells–Riley approach with constant, average ventilation rate $\bar{Q}=r/\gamma$) and the analytical mean infection risk $E[P_{infection}]$ in Equation (12), versus shape parameter r, for $\bar{Q}=1,100,300,500,750,1000{\;m}^{3}\cdot h^{-1}$.

**FIGURE A3:**
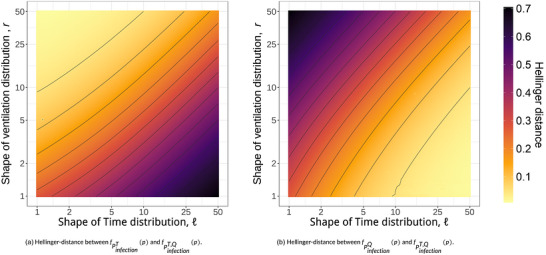
Heatmaps of the Hellinger distance between the density functions of the per capita infection risk probability when two parameters are random ($f_{P_{infection}^{T,Q}}\left(p\right)$) versus when only one is random ($f_{P_{infection}^{T}}\left(p\right)$ or $f_{P_{infection}^{Q}}\left(p\right)$) and the other is set to its mean value instead ($\bar{Q}=r/\gamma$ or $\bar{T}=\ell /\mu$). All default parameter values are taken from the hospitality scenario (Section 3.3.1).

**FIGURE A4:**
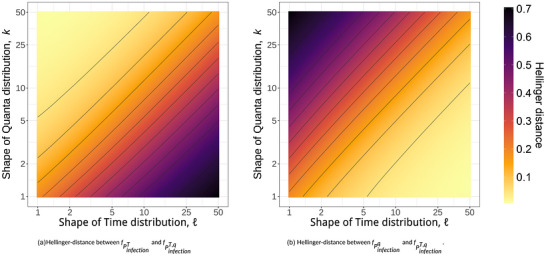
Heatmaps of the Hellinger distance between the density functions of the per capita infection risk probability when two parameters are random ($f_{P_{infection}^{T,q}}\left(p\right)$) versus when only one is random ($f_{P_{infection}^{T}}\left(p\right)$ or $f_{P_{infection}^{q}}\left(p\right)$) and the other is set to its mean value instead ($\bar{q}=k/\lambda$ or $\bar{T}=\ell /\mu$). All default parameter values are taken from the hospitality scenario (Section 3.3.1).

## Appendix

In this Appendix, we detail some of the mathematical derivations omitted in Section 2. The aim is to estimate the density curve and mean of the probability of infection

$$\begin{array}{ccc} P_{infection} & = & 1-e^{-\frac{bqI}{Q}T} \end{array}$$

when one (or two) of the parameters are considered random. We also estimate the probability of observing exactly n exposures during the indoor interaction. In particular, in Appendix A1, we focus on scenarios where a population parameter {b,q,T,I} is random. In Appendix A2, we consider the situation where the ventilation rate Q is random. In Appendix A3, we look at the scenario where a pair of parameters are simultaneously random.

First, let us consider one parameter X∈{b,q,Q,T} being random, and define $g\left(X\right)=-\frac{IbqT}{Q}$, which is then a random variable. Then

$$\begin{array}{ccc} P_{infection} & = & 1-e^{g(X)}\;\Rightarrow \;X=g^{-1}\left(\ln\left(1-P_{infection}\right)\right). \end{array}$$

We can express the cumulative distribution function of $P_{infection}$ in terms of that of *X*

$$\begin{array}{ccc} F_{P_{infection}}\left(p\right) & = & F_{X}\left(g^{-1}\left(\ln\left(1-p\right)\right)\right). \end{array}$$

Now, to obtain the density function of $P_{infection}$, we differentiate $F_{P_{infection}}\left(p\right)$ with respect to p

$$\begin{array}{ccc} \frac{dF_{P_{infection}}\left(p\right)}{dp} & = & \frac{dF_{X}\left(g^{-1}\left(\ln\left(1-p\right)\right)\right)}{dp}, \end{array}$$

from which we obtain

(A1)
$$\begin{array}{ccc} f_{P_{infection}}\left(p\right) & = & f_{X}\left(g^{-1}\left(\ln\left(1-p\right)\right)\right)\left|\frac{dx}{dp}\right|, \end{array}$$

where $f_{P_{infection}}\left(\cdot \right)$ and $f_{X}\left(\cdot \right)$ are the density functions of the random variables $P_{infection}$ and X, respectively. This is known as the change of variables method. To find the expected infection risk, we compute:

(A2)
$$\begin{array}{ccc} E[P_{infection}] & = & \int_{0}^{1}pf_{P_{infection}}\left(p\right)dp\;=\;\int_{0}^{\infty }\left(1-e^{g(x)}\right)f_{X}\left(x\right)dx. \end{array}$$

Finally, since the number of exposures $E∼\text{Binomial}(S,P_{\mathrm{infe}\mathrm{ction}})$, we have

(A3)
P(E=n)=∫0∞Sn1−eg(x)neg(x)S−nfX(x)dx=Sn∑i=0n(−1)ini∫0∞e(S−n+i)g(x)fX(x)dx,

for n=0,⋯,S, where we have used the binomial expansion of ${\left(1-e^{g(x)}\right)}^{n}$.

### A1 Uncertainty in Population Parameters

#### A1.1. Breathing Rate, Quanta Emission Rate or Exposure Time

We consider here a random quanta emission rate q∼Gamma(k,λ) without any loss of generality, since other parameters T and b are symmetric in $P_{infection}$, so analogous results can be obtained. According to the arguments above, we have

$$\begin{array}{cc} g(q)= & -\frac{IbT}{Q}q,\;f_{q}\left(q\right)=\frac{\lambda^{k}q^{k-1}e^{-\lambda q}}{\Gamma (k)}, \end{array}$$

and since $P_{infection}=1-e^{-\frac{IbT}{Q}q}$, one gets $q=g^{-1}\left(\ln\left(1-P_{infection}\right)\right)=\frac{-\ln(1-P_{infection})Q}{IbT}$. Thus,

$$\begin{array}{ccc} \frac{dq}{dP_{infection}} & = & \frac{Q}{(1-P_{infection})IbT} \end{array}$$

and by Equation (A1), the density function of $P_{infection}$ is

(A4)
$$\begin{array}{cc} f_{P_{infection}}\left(p\right)= & \frac{\lambda^{k}{\left(\frac{-\ln(1-p)Q}{IbT}\right)}^{k-1}e^{\left(\frac{\ln(1-p)\;\lambda Q}{IbT}\right)}}{\Gamma (k)}\frac{Q}{(1-p)IbT}\\ = & {\left(\frac{\lambda Q}{IbT}\right)}^{k}\frac{{\left[-\ln\left(1-p\right)\right]}^{k-1}{\left(1-p\right)}^{\frac{\lambda Q}{IbT}-1}}{\Gamma (k)}, \end{array}$$

for p∈(0,1). Using Equation (A2), we find the expectation of $P_{infection}$ as

(A5)
$$\begin{array}{cc} E[P_{infection}]= & \int_{0}^{\infty }\left(1-e^{-\frac{IbT}{Q}q}\right)\frac{\lambda^{k}q^{k-1}e^{-\lambda q}}{\Gamma (k)}dq\\ = & 1-\frac{\lambda^{k}}{\Gamma (k)}\int_{0}^{\infty }e^{-q\left(\frac{IbT}{Q}+\lambda \right)}q^{k-1}dq\\ = & 1-{\left(\frac{\lambda }{\lambda +\frac{IbT}{Q}}\right)}^{k}, \end{array}$$

where we have used

(★)
$$\int_{0}^{\infty }u^{b}e^{-\mathrm{au}}\mathrm{du}=\frac{\Gamma (b+1)}{a^{b+1}},$$

with $a=\frac{IbT}{Q}+\lambda$ and b=k−1. The probability of observing exactly n exposures is computed using Equation (A3) as

(A6)
P(E=n)=Sn∑i=0n(−1)ini∫0∞e−q((S−n+i)IbTQ+λ)qk−1λkΓ(k)dq=Sn∑i=0n(−1)iniΓ(k)(S−n+i)IbTQ+λkλkΓ(k)=Sn∑i=0n(−1)iniλ(S−n+i)IbTQ+λk,

for n=0,⋯,S, where we have again used Equation (★), this time with $a=\left(S-n+i\right)\frac{IbT}{Q}+\lambda$ and b=k−1.

As described in Section 2.1.1, some of the uncertainty in q might arise from mitigations such as mask‐wearing. In particular, given a mask‐wearing probability $w_{mask}$, and the mask's efficacy parameter η, the distribution for the effective quanta emission rate is given by

$$\begin{array}{ccc} f_{q_{eff}}\left(q\right) & = & P\left(mask\right)f_{q_{eff}|\text{mask}}\left(q\right)+P\left(no\;mask\right)f_{q_{eff}|\text{no mask}}\left(q\right)\\ & = & w_{mask}\frac{1}{1-\eta }f_{q_{no\;mask}}\left(\frac{q}{1-\eta }\right)+\left(1-w_{mask}\right)f_{q_{no\;mask}}\left(q\right) \end{array}$$

with $w_{mask}\in \left[0,1\right]$, $f_{q_{no\;mask}}∼\text{Gamma}\left(k,\lambda \right)$ and where $P_{infection}=1-e^{-\frac{Ibq_{eff}T}{Q}}$. In this scenario, our arguments above can be easily adapted. In particular, the density of $P_{infection}$ is given by Equation (A1). By considering a linear combination for $q_{eff}$, only the density for the quanta emission rate changes, not the transformation to $P_{infection}$. Thus, we still have $q=g^{-1}\left(\ln\left(1-P_{infection}\right)\right)=\frac{-\ln(1-P_{infection})Q}{IbT}$. This results in the following infection risk density:

(A7)
$$\begin{array}{ccc} f_{P_{infection}}\left(p\right) & = & \left(\frac{w_{mask}}{1-\eta }f_{q_{no\;mask}}\left(-\frac{\ln(1-p)\;Q}{IbT(1-\eta )}\right)+\left(1-w_{mask}\right)f_{q_{no\;mask}}\left(-\frac{\ln(1-p)\;Q}{IbT}\right)\right)\frac{dq}{dp}\\ & = & \frac{{\left(-\ln\left(1-p\right)\right)}^{k-1}}{\Gamma (k)}{\left(\frac{\lambda Q}{IbT}\right)}^{k}\left(\frac{w_{mask}}{{\left(1-\eta \right)}^{k}}{\left(1-p\right)}^{\frac{\lambda Q}{IbT(1-\eta )}-1}+\left(1-w_{mask}\right){\left(1-p\right)}^{\frac{\lambda Q}{IbT}-1}\right), \end{array}$$

for p∈(0,1). The average infection risk is

(A8)
$$\begin{array}{ccc} E[P_{infection}] & = & \int_{0}^{\infty }\left(1-e^{-\frac{IbqT}{Q}}\right)f_{q_{eff}}\left(q\right)dq\\ & = & 1-\left[w_{mask}{\left(\frac{\lambda }{\lambda +\frac{IbT}{Q}\left(1-\eta \right)}\right)}^{k}+\left(1-w_{mask}\right){\left(\frac{\lambda }{\lambda +\frac{IbT}{Q}}\right)}^{k}\right], \end{array}$$

and the probability of observing exactly n exposures is

(A9)
P(E=n)=Sn∑i=0n(−1)ini∫0∞e(S−n+i)g(q)fqeff(q)dq=Sn∑i=0n(−1)iniwmaskλλ+(S−n+i)IbTQ(1−η)k+(1−wmask)λλ+(S−n+i)IbTQk

for n∈{0,1,⋯,S}. The results given by Equations (A7)–(A9) and their derivations are analogous to that of Equations (A4)–(A6), with the additional term and coefficients.

#### A1.2. Disease Prevalence

If the number of infectors is a discrete random variable I∼Binomial(N,ρ), the corresponding per‐capita probability of infection, $P_{infection}$, is also a discrete random variable (i.e., will take a number of discrete values with different probabilities). In particular, since

P(I=i)=Niρi(1−ρ)N−i,i∈{0,1,⋯,N},

the per‐capita probability of infection $P_{infection}$ takes the form of $1-e^{-\frac{bqT}{Q}i}$ weighted with binomial probabilities P(I=i) for i∈{0,1,⋯,N}

fPinfection(p)=Niρi(1−ρ)N−i,ifp=1−e−bqTQi,0,otherwise.

The expectation of $P_{infection}$ is characterized by:

E[Pinfection]=∑i=0NNiρi(1−ρ)N−i1−e−ibqTQ=1−∑i=0NNiρe−bqTQi(1−ρ)N−i=1−ρe−bqTQ+(1−ρ)N.

The probability of observing exactly n exposures is

P(E=n)=∑i=0N−nP(E=n|I=i)P(I=i)=Nn∑i=0N−nN−ni1−e−bqTQine−bqTQi(N−i−n)ρi(1−ρ)N−i,

for n=0,1,⋯,N.

### A2 Uncertainty in Ventilation Rate

Let us consider Q∼Gamma(r,γ) so that

$$\begin{array}{cc} g(Q)= & -\frac{IbqT}{Q},\;f_{Q}\left(\omega \right)=\frac{\gamma^{r}\omega^{r-1}e^{-\gamma \omega }}{\Gamma (r)}, \end{array}$$

and since $P_{infection}=1-e^{-\frac{IbqT}{Q}}$, one gets $Q=g^{-1}\left(\ln\left(1-P_{infection}\right)\right)=\frac{-IbqT}{\ln(1-P_{infection})}$. Thus,

$$\begin{array}{cc} \frac{dQ}{dP_{infection}}= & \frac{-IbqT}{\left(1-P_{infection}\right){\left(\ln\left(1-P_{infection}\right)\right)}^{2}}. \end{array}$$

Despite this derivative being negative, we use its absolute value in calculating the density function of $P_{infection}$, as according to Equation (A1)

$$\begin{array}{cc} f_{P_{infection}}\left(p\right)= & \frac{\gamma^{r}{\left(\frac{-IbqT}{\ln(1-p)}\right)}^{r-1}e^{\frac{\gamma IbqT}{\ln(1-p)}}}{\Gamma (r)}\frac{IbqT}{\left(1-p\right){\left(\ln\left(1-p\right)\right)}^{2}}\\ = & \frac{{\left(\gamma IbqT\right)}^{r}}{\Gamma (r)}\frac{e^{\frac{\gamma IbqT}{\ln(1-p)}}}{\left(1-p\right){\left[-\ln\left(1-p\right)\right]}^{r+1}},\;p\in \left(0,1\right). \end{array}$$

Using Equation (A2), we find the expectation of $P_{infection}$ as

$$\begin{array}{cc} E[P_{infection}]= & \int_{0}^{\infty }\left(1-e^{-\frac{IbqT}{\omega }}\right)\frac{\gamma^{r}\omega^{r-1}e^{-\gamma \omega }}{\Gamma (r)}d\omega \\ = & 1-\frac{\gamma^{r}}{\Gamma (r)}\int_{0}^{\infty }e^{-\gamma \omega -\frac{IbqT}{\omega }}\omega^{r-1}d\omega \\ = & 1-\frac{1}{\Gamma (r)}\int_{0}^{\infty }e^{-u-\frac{\gamma IbqT}{u}}u^{r-1}du\\ = & 1-\frac{2}{\Gamma (r)}{\left(\gamma IbqT\right)}^{r/2}K_{r}\left(2\sqrt{\gamma IbqT}\right), \end{array}$$

where we used the substitution u=γω and (NIST 2025a)

(†)
$$K_{\theta }\left(\zeta \right)=\frac{1}{2}{\left(\frac{\zeta }{2}\right)}^{\theta }\int_{0}^{\infty }u^{-(\theta +1)}e^{\left(-u-\frac{\zeta^{2}}{4u}\right)}du,$$

with θ=−r and $\zeta =2\sqrt{\gamma IbqT}$, alongside Proposition 2.1 from Glasser et al. (2012) which states $K_{-\theta }\left(\zeta \right)=K_{\theta }\left(\zeta \right)$. The probability of observing exactly n exposures is computed using Equation (A3) as

P(E=n)=Sn∑i=0n(−1)ini∫0∞e(S−n+i)g(ω)fq(ω)dω=Sn∑i=0n(−1)ini∫0∞e−γω−(S−n+i)IbqTωωr−1γrΓ(r)dω=Sn∑i=0n(−1)ini∫0∞e−u−(S−n+i)γIbqTuur−11Γ(r)du(using Equation(†))=2Γ(r)Sn∑i=0n(−1)iniair/2Kr2ai,

for n=0,⋯,S, where $a_{i}=\gamma IbqT\left(S-n+i\right)$ and u=γω.

#### A2.1. Accounting for Mechanical Ventilation

In Section 3.2.3, we look at increasing natural ventilation, $Q_{\mathrm{nat}}∼\text{Gamma}\left(r,\gamma \right)$, by a constant mechanical air change rate, $Q_{mech}=ACH_{mech}\times V$ for mechanical air change rate $ACH_{mech}$ and room volume V. To implement this in our results, we use the derivation from Appendix A2, instead setting $g(Q_{\mathrm{nat}})=-\frac{\mathrm{IbqT}}{Q_{\mathrm{nat}}+Q_{\mathrm{mech}}}$. This means we are now considering $P_{\mathrm{infe}\mathrm{ction}}=1-e^{-\frac{\mathrm{IbqT}}{Q_{\mathrm{nat}}+Q_{\mathrm{mech}}}}$ and $Q_{\mathrm{nat}}=g^{-1}(\ln(1-P_{\mathrm{infe}\mathrm{ction}}))=\frac{-\mathrm{IbqT}}{\ln(1-P_{\mathrm{infe}\mathrm{ction}})}-Q_{\mathrm{mech}}$. The new density function for $P_{infection}$ is

(A10)
$$\begin{array}{cc} f_{P_{\mathrm{infe}\mathrm{ction}}}(p)= & \frac{\gamma^{r}{\left(\frac{-\mathrm{IbqT}}{\ln(1-p)}-Q_{\mathrm{mech}}\right)}^{r-1}e^{\frac{\gamma \mathrm{IbqT}}{\ln(1-p)}+\gamma Q_{\mathrm{mech}}}\mathrm{IbqT}}{\Gamma (r)(1-p)(\ln{\left(1-p)\right)}^{2}},\;p\in \left(0,1-e^{-\frac{\mathrm{IbqT}}{Q_{\mathrm{mech}}}}\right). \end{array}$$

An analytical expression for the mean infection risk, $E[P_{infection}]$, has not been obtained in this paper. The introduction of $Q_{mech}$ increases the complexity of the underlying integral. The mean infection risk values for Figure 10b,d were obtained numerically instead.

### A3 Uncertainty in Two Model Parameters

Let us consider the situation here where two model parameters are random. Here, we consider two population‐related parameters in {b,q,T} being (independently) random; say q∼Gamma(k,λ) and T∼Gamma(ℓ,μ) without any loss of generality. Given that q and T are independent, their joint density is $f_{q,T}\left(q,t\right)=f_{q}\left(q\right)f_{T}\left(t\right)$. Using the change of variables method, we can write

$$\begin{array}{cc} f_{Z,T}\left(z,t\right)= & f_{T}\left(t\right)f_{q}\left(z/t\right)\frac{dq}{dz}\;=\;f_{T}\left(t\right)f_{q}\left(z/t\right)\frac{1}{t}, \end{array}$$

where Z=qT. We then integrate this joint density over possible values of T to get:

$$\begin{array}{cc} f_{Z}\left(z\right)= & \int_{0}^{\infty }t^{-1}f_{T}\left(t\right)f_{q}\left(z/t\right)dt=\int_{0}^{\infty }t^{-1}\frac{\mu^{\ell }t^{\ell -1}e^{-\mu t}}{\Gamma (\ell )}\frac{\lambda^{k}{\left(\frac{z}{t}\right)}^{k-1}e^{-\frac{\lambda z}{t}}}{\Gamma (k)}dt\\ = & \frac{\mu^{k}\lambda^{k}z^{k-1}}{\Gamma (\ell )\Gamma (k)}\int_{0}^{\infty }u^{\ell -k-1}e^{-u-\frac{{\left(2\sqrt{\lambda \mu z}\right)}^{2}}{4u}}du\\ = & \frac{2{\left(\lambda \mu z\right)}^{\frac{1}{2}\left(k+\ell \right)}K_{k-\ell }\left(2\sqrt{\lambda \mu z}\right)}{z\Gamma (\ell )\Gamma (k)}, \end{array}$$

where we used the substitution u=μt and Equation (†) with r=k−ℓ and $\zeta =\sqrt{\lambda \mu z}$. So, we have

$$\begin{array}{c} g\left(Z\right)=-\frac{Ib}{Q}Z,\;f_{Z}\left(z\right)=\frac{2{\left(\lambda \mu z\right)}^{\frac{1}{2}\left(k+\ell \right)}K_{k-\ell }\left(2\sqrt{\lambda \mu z}\right)}{z\Gamma (\ell )\Gamma (k)}, \end{array}$$

where $P_{infection}=1-e^{-\frac{Ib}{Q}Z}$ results in $Z=g^{-1}\left(\ln\left(1-P_{infection}\right)\right)=\frac{-\ln(1-P_{infection})Q}{Ib}$. Therefore,

$$\begin{array}{c} \frac{dZ}{dP_{infection}}=\frac{Q}{Ib(1-P_{infection})} \end{array}$$

and the resulting density function of $P_{infection}$ is

$$\begin{array}{cc} f_{P_{infection}}\left(p\right)= & \frac{2{\left(\lambda \mu \left(\frac{-\ln(1-p)Q}{Ib}\right)\right)}^{\frac{1}{2}\left(k+\ell \right)}K_{k-\ell }\left(2\sqrt{\lambda \mu \frac{-\ln(1-p)Q}{Ib}}\right)}{\frac{-\ln(1-p)Q}{Ib}\Gamma \left(\ell \right)\Gamma \left(k\right)}\frac{Q}{Ib(1-p)}\\ = & \frac{2{\left(\lambda \mu \right)}^{\frac{k+\ell }{2}}{\left[-\frac{1}{c}\ln\left(1-p\right)\right]}^{\frac{k+\ell }{2}-1}K_{k-\ell }\left(2\sqrt{d\left[-\ln(1-p)\right]}\right)}{c\Gamma (k)\Gamma (\ell )(1-p)} \end{array}$$

with $c=\frac{Ib}{Q}$, $d=\frac{\lambda \mu }{c}$ and for p∈(0,1). The expected per‐capita infection risk is

(A11)
$$\begin{array}{cc} E[P_{infection}]= & \int_{0}^{\infty }\int_{0}^{\infty }\left(1-e^{-\frac{Ibqt}{Q}}\right)f_{q}\left(q\right)f_{T}\left(t\right)dqdt\\ = & 1-\int_{0}^{\infty }\frac{\lambda^{k}\mu^{\ell }}{\Gamma (k)\Gamma (\ell )}q^{k-1}e^{-\lambda q}\frac{\Gamma (\ell )}{{\left(\mu +\frac{Ibq}{Q}\right)}^{\ell }}dq\;\left(\text{using Equation}\left(★\right)\right)\\ = & 1-{\left(\frac{\lambda \mu Q}{Ib}\right)}^{k}\frac{1}{\Gamma (k)}\int_{0}^{\infty }e^{-\frac{\lambda \mu Q}{Ib}v}v^{k-1}{\left(1+v\right)}^{-\ell }dv\\ = & 1-d^{k}U\left(k,k-\ell +1,d\right), \end{array}$$

where we have used $v=\frac{Ib}{\mu Q}q$, and U(a,b,z) is the confluent hypergeometric function of the second kind (NIST 2025c), sometimes referred to as the *Tricomi* function

(‡)
$$\begin{array}{c} U\left(a,b,z\right)=\frac{1}{\Gamma (a)}\int_{0}^{\infty }e^{-zv}v^{a-1}{\left(1+v\right)}^{b-a-1}dv. \end{array}$$

By solving the double integral with respect to q first, or using Kummer's transformation (NIST 2025e)

(★★)
$$\begin{array}{c} U\left(a,b,z\right)=z^{1-b}U\left(1+a-b,2-b,z\right), \end{array}$$

an alternate (equivalent) form for the expected per‐capita infection risk can be obtained:

$$\begin{array}{c} E\left[P_{infection}\right]=1-d^{\ell }U\left(\ell ,\ell -k+1,d\right). \end{array}$$

The probability of observing exactly n exposures is given by

(A12)
P(E=n)=Sn∑i=0n(−1)ini∫0∞∫0∞e−(S−n+i)IbqtQfq(q)fT(t)dqdt,=Sn∑i=0n(−1)ini∫0∞λkμℓΓ(k)Γ(ℓ)qk−1e−λqΓ(ℓ)(μ+IbQ(S−n+i)q)ℓdq,(using Equation(★))=Sn∑i=0n(−1)iniλμQIb(S−n+i)k1Γ(k)∫0∞e−λμQIb(S−n+i)vvk−1(1+v)−ℓdv=Sn∑i=0n(−1)iniyikU(k,k−ℓ+1,yi),(using Equation(‡))

for n=0,⋯,S, where we have used the substitution $v=\frac{Ib}{\mu Q}q\left(S-n+i\right)$ and parameter $y_{i}=d/\left(S-n+i\right)$. By solving the double integral with respect to q first, or using Kummer's transformation (Equation (★★)) an alternate (equivalent) form for the n‐exposure probability can be obtained

P(E=n)=Sn∑i=0n(−1)iniyiℓU(ℓ,ℓ−k+1,yi),

for n=0,⋯,S. We note that if one of the parameters is Erlang‐distributed, where the shape parameter is a positive integer, Equations (A11) and (A12) may be written as finite sums of upper incomplete gamma functions. For example, if k∈N so that q is Erlang‐distributed, one gets

E[P]=∫0∞∫0∞1−e−IbqtQfq(q)fT(t)dqdt=∫0∞1−μμ+IbqQℓλkqk−1e−λq(k−1)!dq=1−λkμℓ(k−1)!∫λμQIb∞w−λμQIbk−1λ1−ke−w+λμQIbwℓIbλQℓ1λdw=1−eλμQIb(k−1)!∑i=0∞k−1i(−1)iλμQIbi+ℓ∫λμQIb∞wk−ℓ−i−1e−wdw,=1−ed(k−1)!∑i=0k−1k−1i(−1)idi+ℓΓk−ℓ−i,d,

where $w=\lambda q+\frac{\lambda \mu Q}{Ib}$ and Γ(s,x) is the upper incomplete gamma function (NIST 2025f)

(‡‡)
$$\begin{array}{c} \Gamma \left(s,x\right)=\int_{x}^{\infty }w^{s-1}e^{-w}dw. \end{array}$$

Similarly, one gets

P(E=n)=Sn∑i=0n∑j=0k−1(−1)i+j(k−1)!nik−1jyiℓ+jeyiΓ(k−ℓ−j,yi),

for n=0,⋯,S. Similar arguments apply when ℓ∈N, just by swapping k with ℓ and λ for μ in each derivation.

Finally, one can consider one random population‐related parameter in {b,q,T} and one random ventilation parameter (Q). Because Q appears on the denominator of the product of the exponent in the classical Wells–Riley model, new equations for $P_{infection}$ arise. Let us assume that q is random without loss of generality, as the methods will be identical for b or T, so that q∼Gamma(k,λ) and Q∼Gamma(r,γ). The quotient of two Gamma distributions is known to result in a Generalised Beta Prime distribution. In particular, for Y=q/Q we have $Y∼\beta^{\prime }\left(k,r,1,\frac{\gamma }{\lambda }\right)$, with density function

$$\begin{array}{c} f_{Y}\left(y\right)=\frac{\lambda^{k}\gamma^{r}y^{k-1}\Gamma \left(k+r\right)}{\Gamma \left(k\right)\Gamma \left(r\right){\left(\lambda y+\gamma \right)}^{k+r}}. \end{array}$$

We now have,

$$\begin{array}{c} g(Y)=-IbTY. \end{array}$$

Moreover, $Y=g^{-1}\left(\ln\left(1-P_{infection}\right)\right)=-\frac{\ln(1-P_{infection})}{IbT}$, which means

$$\begin{array}{c} \frac{dY}{dP_{infection}}=\frac{1}{(1-P_{infection})IbT}. \end{array}$$

Using Equation (A1), we find the density

$$\begin{array}{cc} f_{P_{infection}}\left(p\right)= & \frac{\lambda^{k}\gamma^{r}{\left[-\frac{\ln(1-p)}{IbT}\right]}^{k-1}\Gamma \left(k+r\right)}{\Gamma \left(k\right)\Gamma \left(r\right){\left(\lambda \left[-\frac{\ln(1-p)}{IbT}\right]+\gamma \right)}^{k+r}}\frac{1}{(1-p)IbT}\\ = & \frac{\Gamma (k+r)}{\Gamma (k)\Gamma (r)}\frac{\lambda^{k}{\left(\gamma IbT\right)}^{r}{\left[-\ln\left(1-p\right)\right]}^{k-1}}{\left(1-p\right){\left(\left[-\ln\left(1-p\right)\right]\lambda +\gamma IbT\right)}^{k+r}}, \end{array}$$

for p∈(0,1). The expected per‐capita infection risk is

(A13)
$$\begin{array}{cc} E[P_{infection}]= & \;\int_{0}^{\infty }\int_{0}^{\infty }\left(1-e^{-\frac{IbqT}{\omega }}\right)f_{q}\left(q\right)f_{Q}\left(\omega \right)dqd\omega \\ = & \;1-\int_{0}^{\infty }\frac{\lambda^{k}\gamma^{r}}{\Gamma (k)\Gamma (r)}\omega^{r-1}e^{-\gamma \omega }\frac{\Gamma (k)}{{\left(\lambda +\frac{IbT}{\omega }\right)}^{k}}d\omega \;\left(\text{using Equation}\;\left(★\right)\right)\\ = & \;1-{\left(\frac{\gamma IbT}{\lambda }\right)}^{r}\frac{1}{\Gamma (r)}\int_{0}^{\infty }e^{-\frac{\gamma IbT}{\lambda }}v^{k+r-1}{\left(1+v\right)}^{-k}dv\\ = & \;1-{\left(\frac{\gamma IbT}{\lambda }\right)}^{r}\frac{\Gamma (k+r)}{\Gamma (r)}U\left(k+r,r+1,\frac{\gamma IbT}{\lambda }\right),\;\left(\text{using Equation}\;\left(‡\right)\right) \end{array}$$

where we have used $v=\frac{\lambda }{IbT}\omega$. The probability of observing exactly n exposures is

(A14)
P(E=n)=Sn∑i=0n(−1)ini∫0∞∫0∞e−(S−n+i)IbqTωfq(q)fQ(ω)dqdω=Sn∑i=0n(−1)ini∫0∞λkγrΓ(k)Γ(r)ωr−1e−γωΓ(k)(λ+IbTω(S−n+i))kdω,(using Equation(★))=Sn∑i=0n(−1)iniγIbTλ(S−n+i)r1Γ(r)∫0∞e−γIbTλ(S−n+i)vk+r−1(1+v)−kdv=Sn∑i=0nni(−1)iαirΓ(k+r)Γ(r)Uk+r,r+1,αi,(w/ Equation(‡))

for n=0,⋯,S and $\alpha_{i}=\frac{\gamma IbT}{\lambda }\left(S-n+i\right)$. Here we have used Equation (★) and the substitution $v=\frac{\lambda }{IbT(S-n+i)}\omega$. Equations (A13) and (A14) have simplified forms when some of the parameters are Erlang‐distributed. For example, if both k,r∈N, we have

E[Pinfection]=∫0∞∫0∞1−e−IbqTωfq(q)fQ(ω)dqdω,=1−λkγrΓ(r)∫0∞ωr−1e−γωγω+γIbTλkλγωkdω,(using Eq.(★))=1−eγIbTλΓ(r)∫γIbTλ∞(w−γIbTλ)r+k−1w−ke−wdw=1−eγIbTλΓ(r)∑i=0r+k−1r+k−1i(−1)iγIbTλi∫γIbTλ∞wr−1−ie−wdw=1−eγIbTλ(r−1)!∑i=0r+k−1r+k−1i(−1)iγIbTλiΓr−i,γIbTλ

which uses the substitution $w=\gamma \omega +\frac{\gamma IbT}{\lambda }$ and Equation (‡‡). The probability of observing exactly n exposures simplifies to

P(E=n)=Sn∑i=0n(−1)ini∫0∞∫0∞e−(S−n+i)IbqTωfq(q)fQ(ω)dydω=Sn∑i=0n(−1)ini∫0∞λkγrΓ(k)Γ(r)ωr−1e−γωΓ(k)(λ+IbTω(S−n+i))kdω=Sn∑i=0n(−1)iniλkγrΓ(r)∫0∞ωr−1(γω+γIbTλ(S−n+i))−k(γω)kλ−ke−γωdω=Sn1(r−1)!∑i=0n∑j=0r+k−1(−1)i+jnir+k−1jαijeαi∫αi∞wr−1−je−wdw=Sn1(r−1)!∑i=0n∑j=0r+k−1(−1)i+jnir+k−1jαijeαiΓ(r−j,αi),

for n=0,⋯,S, where we used the substitution $w=\gamma \omega +\alpha_{i}$ and Equation (‡‡).

### A4 Population Heterogeneity

When considering a random quanta generation rate q∼Gamma(k,λ), it is important to note that if I>1 and one still considers qI in the corresponding analysis, then the main assumption is that all infectors have the same quanta generation rate q (which is sampled *once* from the corresponding distribution Gamma(k,λ)). This would be appropriate for situations where one wants to consider homogeneous infectiousness across infectors but uncertainty in the precise value of q. For situations where I>1, and if the aim is to consider heterogeneity in infectiousness, it is more appropriate to consider that each individual i∈{1,2,⋯,I} has quanta generation rate $q_{i}∼\text{Gamma}(k,\lambda )$, where this Gamma distribution represents population‐level heterogeneity in infectiousness. In this situation, the total quanta emission rate is given by the sum of rates from each individual: $q_{Total}=\sum_{i=1}^{I}q_{i}$. Since the sum of I independent Gamma distributions with shape k and rate λ is itself a Gamma distribution of shape kI and rate λ, our results can be easily generalized to this scenario; that is, $q_{Total}∼\text{Gamma}\left(Ik,\lambda \right)$ and we consider $P_{infection}=1-e^{-\frac{bT}{Q}q_{Total}}$. In particular, one has

$$\begin{array}{c} g\left(q_{Total}\right)=-\frac{bT}{Q}q_{Total},\;f_{q_{Total}}\left(q\right)=\frac{\lambda^{Ik}q^{Ik-1}e^{-\lambda q}}{\Gamma (Ik)}. \end{array}$$

Since $P_{infection}=1-e^{\frac{-bT}{Q}q_{Total}}$ we have $q_{Total}=\frac{-\ln(1-P_{infection})Q}{bT}$ and

$$\begin{array}{c} \frac{dq_{Total}}{dP_{infection}}=\frac{Q}{(1-P_{infection})bT}. \end{array}$$

Thus, Equation (A1) results in the density function of $P_{infection}$

(A15)
$$\begin{array}{cc} f_{P_{infection}}\left(p\right)= & \frac{\lambda^{Ik}{\left[\frac{-\ln(1-p)Q}{bT}\right]}^{Ik-1}e^{-\lambda \left[\frac{-\ln(1-p)Q}{bT}\right]}}{\Gamma (Ik)}\frac{Q}{(1-p)bT}\\ = & {\left(\frac{\lambda Q}{bT}\right)}^{Ik}\frac{{\left(-\ln(1-p)\right)}^{Ik-1}{\left(1-p\right)}^{\frac{\lambda Q}{bT}-1}}{\Gamma (Ik)},\;p\in \left(0,1\right). \end{array}$$

Using Equation (A2), we find the expected per‐capita infection risk

(A16)
$$\begin{array}{cc} E[P_{infection}]= & \int_{0}^{\infty }\left(1-e^{-\frac{bT}{Q}q}\right)\frac{\lambda^{Ik}q^{Ik-1}e^{-\lambda q}}{\Gamma (Ik)}dq\\ = & 1-\frac{\lambda^{Ik}}{\Gamma (Ik)}\int_{0}^{\infty }e^{-q\left(\frac{bT}{Q}+\lambda \right)}q^{Ik-1}dq\;=\;1-{\left(\frac{\lambda }{\frac{bT}{Q}+\lambda }\right)}^{Ik}, \end{array}$$

where we have used Equation (★). The probability of observing exactly n exposures is computed using Equation (A3) leading to

(A17)
P(E=n)=Sn∑i=0n(−1)ini∫0∞e−q((S−n+i)bTQ+λ)qIk−1λIkΓ(Ik)dq=Sn∑i=0n(−1)iniΓ(Ik)(S−n+i)bTQ+λIkλIkΓ(Ik)=Sn∑i=0n(−1)iniλ(S−n+i)bTQ+λIk,

for n=0,⋯,S.

Due to the symmetry in the product in the equation for the classical Wells–Riley model, these results are analogous to the case of sequential visits, such that we have a sum of M independent randomly distributed exposure times, $T_{Total}=\sum_{i=1}^{M}T_{i}$, instead of a sum of quanta emission rates. This is the approach used in Section 3.2.1 and Figure 8 in particular.

### A5 Comparing to the Classical Wells–Riley Approach

Here, we aim to compare the “true” average risk $E[P_{infection}]$ estimated via our approach with the per capita infection risk estimated using the classical Wells–Riley methodology with average parameter value instead, ${\bar{P}}_{infection}^{WR}$.

#### A5.1. Random Population Parameter

For a single random population parameter, one can estimate the average per capita infection risk $E[P_{infection}]$ using Equation (3). It is of interest to compare this “true” mean risk value with that obtained via the classical Wells–Riley approach in Equation (1) when using an average quanta emission rate $\bar{q}=k/\lambda$. In Section 2.1.1, we denote this by

$$\begin{array}{c} {\bar{P}}_{infection}^{WR}=1-e^{-\frac{IbT}{Q}\bar{q}}. \end{array}$$

It is of particular interest to analyze the difference between the resulting average infection risk of each approach. We can denote this difference by

(A18)
$$\begin{array}{cc} D(A,k,\lambda )= & {\bar{P}}_{infection}^{WR}-E\left[P_{infection}\right]={\left(\frac{\lambda }{\lambda +A}\right)}^{k}-e^{-A\frac{k}{\lambda }}, \end{array}$$

where A represents the combination of non‐random parameters, such that $g\left(X\right)=-\frac{IbqT}{Q}=-AX$ for some random parameter X∈{I,b,q,T} (e.g., $A=\frac{IbT}{Q}$ for random q), and (k,λ) represent the shape and rate parameters of the Gamma‐distributed random parameter. Depending on the sign of D(A,k,λ), we can make the following conclusions:

- D(A,k,λ)>0: The classical approach with average parameter value is overestimating the “true” mean infection risk.
- D(A,k,λ)=0 (or ≈0): The classical approach with average parameter value is capturing the “true” mean infection risk.
- D(A,k,λ)<0: The classical approach with average parameter value is underestimating the “true” mean infection risk.

In the case of random b,q, or T one can use Jensen's inequality to show that the classical approach (${\bar{P}}_{infection}^{WR}=1-e^{A\bar{X}}$) will always overestimate the true mean infection risk, $E[P_{infection}]$. Jensen's inequality (Jensen 1906; McShane 1937) states that, for a strictly concave function P(q) (sometimes referred to as “concave‐down”) and a nonconstant random variable q, the following inequality holds:

$$\begin{array}{c} E[P(q)]<P(E[q]). \end{array}$$

The per‐capita infection probability $P_{infection}\left(q\right)=1-e^{-Aq}$ is a concave function of q since the second derivative is always negative,

$$\begin{array}{c} \frac{d^{2}P_{infection}}{dq^{2}}=-A^{2}e^{-Aq}<0. \end{array}$$

So, we recognize $P(E[q])={\bar{P}}_{\mathrm{infe}\mathrm{ction}}^{\mathrm{WR}}$, and $E\left[P\left(q\right)\right]=E\left[P_{infection}\right]$, to conclude that

$$\begin{array}{c} D\left(A,k,\lambda \right)={\bar{P}}_{infection}^{WR}-E\left[P_{infection}\right]>0 \end{array}$$

holds for all A>0 and k,λ. Furthermore, since $P_{infection}$ is always concave this result holds for generally distributed b,q, or T. So, given A>0 and randomly distributed b,q, or T, the classical Wells–Riley approach (which uses the mean value of the random parameter) will always overestimate the true mean infection risk, $E[P_{infection}]$.

If we fix A and the mean $\bar{q}$, then we can write D(A,k,λ)=D(k), since $\lambda =k/\bar{q}$ (now depends on k). Then

$$\begin{array}{cc} D(k)= & {\bar{P}}_{infection}^{WR}-E\left[P_{infection}\right]={\left(\frac{k}{k+A\bar{q}}\right)}^{k}-e^{-A\bar{q}}. \end{array}$$

By taking the derivative with respect to k, we can find the value $k_{max}$ at which we have the largest overestimation:

(A19)
$$\begin{array}{c} \frac{dD(k)}{dk}={\left(\frac{k}{k+A\bar{q}}\right)}^{k}\left[\ln\left(\frac{k}{k+A\bar{q}}\right)+1-\frac{k}{k+A\bar{q}}\right], \end{array}$$

for which no finite k satisfies $\frac{dD(k)}{dk}=0$ (since we would need $\frac{k}{k+A\bar{q}}=1$ which only holds in the limit of k→∞). We also find that $\frac{dD(k)}{dk}<0$ for all finite k>0, meaning that D(k) is monotonically decreasing. This means that for fixed A, and fixed mean $\bar{q}$, the largest overestimation appears when k→0 (i.e., for small values of the shape parameter in the Gamma distribution for q). We can determine the maximum overestimation for any fixed mean $\bar{q}=k/\lambda$. Using a logarithmic argument, one can find $\lim_{k\to 0^{+}}D\left(k\right)=1-e^{-A\bar{q}}={\bar{P}}_{infection}^{WR}$. This means that as the mean $\bar{q}$ and *A* increase (decrease), the maximum possible overestimation increases (decreases), and this maximum occurs in the limit k→0 (implying λ→0 since $\bar{q}$ is fixed). As k and λ both decrease such that the mean, $\bar{q}=k/\lambda$, is fixed, two things happen:

1. The variance, $\frac{k}{\lambda^{2}}=\frac{\bar{q}}{\lambda }$, of the random parameter increases.
2. The skewness, $\frac{2}{\sqrt{k}}$, increases.

We can conclude that large variance and positive skewness of a random population parameter leads to larger overestimations of the density mean by the classical Wells–Riley model (with mean parameter value), where all the other parameters are fixed. This could suggest that inaccuracies are caused by the fact that considering the mean value of our random population parameter neglects small values (with non‐negligible probabilities) which significantly impact infection risk, particularly in the case of a high variance and positively skewed Gamma distribution. When we have a distribution that is highly concentrated around the mean with little skewness (when k is very high), then the overestimation becomes small (one can show that $\lim_{k\to \infty }D\left(k\right)=0$). It should be noted that the maximum overestimation is affected by the value of A, if A≪1 (A≫1), then the density mean, $E[P_{infection}]$, and the classical Wells–Riley, ${\bar{P}}_{infection}^{WR}$, will both approach zero (one), where their difference disappears.

Let us consider some numerical experiments to explore this further. We focus here on the parameter q but a similar analysis would apply to T or b if they were Gamma‐distributed. We consider the quanta generation rate to follow a Gamma distribution

$$\begin{array}{ccc} q & ∼ & \text{Gamma}(k,\lambda ). \end{array}$$

The aim is to explore how different combinations of (k,λ) impact on the per capita infection risk and the error of the classical approach relative to the analytical mean infection risk: $100\times D(A,k,\lambda )/E[P_{\mathrm{infe}\mathrm{ction}}]\;\%$. To this end, we uniformly sample b, T, and Q, as well as the shape, k, and rate, λ, parameters of the Gamma distribution describing q. We use wide intervals that produce plausible values for the Wells–Riley parameters and a wide range of variances for q. In particular, we choose $\bar{q}\in \left[0.01,100\right]\;\mathrm{quanta}\;h^{-1}$ and k∈[1,20] (such that $\lambda =k/\bar{q}$). Likewise, for the fixed parameters (sampled here but treated as fixed after the fact, as in Equation (3)) we consider: $b\in \left[0.2,0.6\right]\;m^{3}h^{-1}$, T∈[5min,100h], and $Q\in \left[10,1000\right]\;m^{3}h^{-1}$ (which represents ACH∈[0.1,10] in a typical ward (Edwards 2024)), such that I=1. These samples combine to make pairs of A=bT/Q and $\bar{q}=k/\lambda$, for which we plot the corresponding relative errors: $100\times D(A,k,\lambda )/E[P_{\mathrm{infe}\mathrm{ction}}]\;\%$, in Figure 1 within Section 2.1.1. We noticed that overestimations were observed for a “strip” of values $\left(\bar{q},bT/Q\right)$ within the upper right region of Figure 1. Furthermore, we noticed variations points that were adjacent to one another, suggesting that the size of the overestimations do not depend on $\bar{q}$ and bT/Q alone. To investigate this, we plot the relative difference while varying k, the shape parameter of Gamma‐distributed q, and the value of bT/Q for different mean quanta emission rates $\bar{q}$ in Figure A1. By observing the resulting pattern in the relative difference, we can see that (i) the size of the interval of bT/Q is about one order of magnitude on the log scale, and its position is directly dependent on the order of $\bar{q}$, (ii) within this region where overestimations occur, the largest are observed when k is small (1 here); this agrees with our earlier intuition about large variation and positive skewness of q leading to systematic inaccuracies. The dark regions in Figure A1 (indicating little to no relative difference) are those where the infection risk is either close to zero/one (where bT/Q is small/large) or where the shape parameter is so high that there is little uncertainty in the quanta emission rate q around its mean value $\bar{q}$. We identify the following criteria which lead to overestimations by the classical WR approach (${\bar{P}}_{infection}^{WR}$) of the analytical mean infection risk ($E[P_{infection}]$):

1. A mean parameter value and A value ($\bar{q}=k/\lambda$ and bT/Q in this case) that is neither too small nor too large, so as to avoid saturation of the infection risk at either 0 or 1.
2. A large variance and positive skewness for the distribution of the random parameter, such that smaller values (which now have sufficiently high probabilities of occurring) are neglected when considering the mean parameter value.

#### A5.2. Random Ventilation Rate

In the case of a random ventilation rate, the classical Wells–Riley approach is now given by

$$\begin{array}{c} {\bar{P}}_{infection}^{WR}=1-e^{-\frac{IbqT}{\bar{Q}}}, \end{array}$$

as in Section 2.2, where Q∼Gamma(r,γ), meaning $\bar{Q}=\frac{r}{\gamma }$. As such, the difference between the “true” mean infection risk and the mean calculated by the classical Wells–Riley approach with average ventilation rate, is given by

$$\begin{array}{c} D\left(A,r,\gamma \right)=\frac{2}{\Gamma (r)}{\left(\gamma A\right)}^{r/2}K_{r}\left(2\sqrt{\gamma A}\right)-e^{-A\frac{r}{\gamma }}, \end{array}$$

such that A=IbqT. The function $P_{infection}\left(Q\right)=1-e^{-\frac{A}{Q}}$ is neither totally concave or convex. This means that we cannot explicitly apply Jensen's inequality here. However, we can find the inflection point of $P_{infection}$. The second derivative of $P_{infection}$ with respect to Q is

$$\begin{array}{c} \frac{d^{2}P_{infection}\left(Q\right)}{dQ^{2}}=\frac{A}{Q^{3}}e^{-\frac{A}{Q}}\left(2-\frac{A}{Q}\right). \end{array}$$

Setting this to zero and solving for Q tells us that the inflection point is $Q^{*}=A/2$. For $Q\in (0,Q^{*})$, we have $\frac{d^{2}P_{infection}}{dQ^{2}}<0$, and for $Q\in (Q^{*},\infty )$ the opposite is true $\frac{d^{2}P_{infection}}{dQ^{2}}>0$. This means that there are two distinct regions:

- Concave region: $\left(0,Q^{*}\right)$.
- Convex region: $\left(Q^{*},\infty \right)$.

If the support for Q is contained exclusively within the convex (concave) region, then we can conclude, by Jensen's inequality, that the classical Wells–Riley approach would underestimate (overestimate) the true mean infection risk, given by $E[P_{infection}]$.

Using $b\in \left[0.2,0.6\right]m^{3}h^{-1}$, T∈[5min,100h] (as in Appendix A5.2), $q\in \left[0.01,100\right]\;\mathrm{quanta}\;h^{-1}$ (to represent values considered in Section 3), along with $\bar{Q}\in \left[10,1000\right]\;m^{3}h^{-1}$ (to represent ACH∈[0.1,10] for a typical ward (Edwards 2024)) and r∈[1,20] (such that $\gamma =r/\bar{Q}$), we now numerically investigate possible inaccuracies in the case of random ventilation. Combining these samples, we obtain pairs of A=bqT and $\bar{ACH}=\frac{\bar{Q}}{100}$ for which we plot the error of the classical Wells–Riley approach (${\bar{P}}_{infection}^{WR}$) relative to the analytical mean infection risk ($E[P_{infection}]$) in Figure 2 within Section 2.2. First, we notice that, unlike Figure 1, the range of inaccuracies spans both positive and negative values relating to both overestimations and underestimations (with overestimations being very small). For the samples that we generated, the overestimations seem to only occur for very large A=bqT values. Mathematically, we know that this can be caused by the entirety (or large enough portion) of the distribution for Q existing in the concave region of $P_{infection}$, $\left(0,Q^{*}\right)$ where $Q^{*}=A/2$. Intuitively, these overestimations, albeit small, may be due to the fact that using the mean value of Q in place of its distribution neglects higher ventilation rates. It is possible that when A=bqT is large enough, using the mean ventilation rate results in saturation of $P_{infection}$ near 1, but larger and statistically significant ventilation rates would outweigh this saturation if they were to occur. Therefore, in this case, we are neglecting scenarios in which the infection risk does not saturate at 1. This overestimation is likely smaller in magnitude compared to the possible underestimations under different parameter regimes because the Gamma distribution is positively skewed towards smaller values, so the effect of neglecting larger values is dampened as they have smaller probabilities of occurring. Even with a positively skewed distribution, there are scenarios in which the mean does not adequately capture the larger ventilation rates that result in smaller infection risks.

As with the case for random q (see Appendix A5.1), we observe local fluctuations of the relative difference where underestimations are observed in Figure 2, suggesting that $\bar{ACH}$ and bqT alone do not determine the degree of underestimation. We again plot the relative difference for varying bqT, shape parameter of the ventilation rate, r, and for different mean ventilation rates $\bar{Q}$ in Figure A2. First, we notice that a lower bound for the region of underestimations (non‐black color) is not observed for the parameter values considered. However, we know that a lower bound exists by using a limit approach which confirms $\lim_{bqT\to 0}\left({\bar{P}}_{infection}^{WR}\right)=\lim_{bqT\to 0}\left(E\left[P_{infection}\right]\right)$ (implying that D(A,r,γ)→0). So, at least for these parameter values, the region at which we observe relative difference is magnitudes larger in the case of random ventilation than it is for random quanta. Furthermore, the degree of this relative difference is also much larger in the random ventilation case (over 80%), and occurs also for small values of the shape parameter, r, corresponding to high variation and positive skew in Q. We see examples of this of this when comparing the errors seen in Figures 7, 8, 9 to those in Figure 10a,c. Another example is the error produced by only considering random time (green curves) compared to only considering random ventilation (blue curves) for high variance ventilation in Figure 11. The reason for the higher degree of relative difference for random ventilation versus random quanta likely has to do with how sensitive the Wells–Riley model is to their respective fluctuations. That is, if q is random, then the classical approach does not vary as strongly in the region of $\bar{q}$, as it would in the region of $\bar{Q}$ in the case of random Q. When $\bar{Q}$ is random, the possibility of small removal rates causes the infection risk to increase notably, even sometimes to close to 1 (as observed in Figure 10c), whereas the same effect being achieved in the case of random quanta requires the possibility of very large q, which is not as likely in the case of a positively skewed distribution. As with the case for random quanta, when ventilation is random, the following criteria are identified which are required for overestimations to occur:

1. A mean value, $\bar{Q}$, that “balances” the value of the other parameters, A=bqT, is necessary so as to avoid saturation of $P_{infection}$ at 0 or 1.
2. A large enough variance and positive skewness for fixed mean $\bar{Q}$, such that r and γ are small, is necessary by which small values leading to significantly high infection risks are neglected.

### A6 Impact of Two Random Parameters on Infection Risk: Uncertainty Dominance

In this section, the aim is to understand how simultaneous uncertainty in two different parameters may affect infection risk. In particular, there might be situations where even if both parameters are random, there is one that clearly dominates the impact on the per capita infection risk probability $P_{infection}$. Let us consider two random parameters X,Y∈{b,q,T,Q,I} and denote by $P_{infection}^{X,Y}$ the per capita probability of infection when both parameters are considered to be random simultaneously. On the other hand, let us denote by $P_{infection}^{X}$ the per capita probability of infection when X is considered to be random, but parameter Y is considered constant and set instead to its mean value $\bar{Y}$.

Using arguments from Section 2, we can compute the density functions of $P_{infection}^{X,Y}$ and $P_{infection}^{X}$, denoted by $f_{P_{infection}^{X,Y}}\left(p\right)$ and $f_{P_{infection}^{X}}\left(p\right)$, respectively. Results in Section 3.3.1 (Figure 11) suggest that high variance and skewness in the distribution of the random parameters lead to larger propagation of uncertainty into the corresponding infection risk probability. More specifically, if the variance (and skewness) of one random parameter, X, is larger than that of another, Y, we expect this parameter to dominate the propagation of uncertainty into $P_{infection}^{X,Y}$ and, thus, $f_{P_{infection}^{X,Y}}\left(p\right)$. This would mean that the density functions $f_{P_{infection}^{X,Y}}\left(p\right)$ and $f_{P_{infection}^{X}}\left(p\right)$ are more similar to each other, compared to $f_{P_{infection}^{Y}}\left(p\right)$ (i.e., most of the uncertainty in infection risk comes from uncertainty in X, and not in Y). To quantify this, we can make use of the Hellinger distance (Aggarwal and Reddy 2014). The Hellinger distance measures the difference between two distributions, $f_{X}\left(x\right)$ and $g_{X}\left(x\right)$ as

$$\begin{array}{c} H^{2}\left(f_{X},g_{X}\right)=\frac{1}{2}\int {\left(\sqrt{f_{X}\left(x\right)}-\sqrt{g_{X}\left(x\right)}\;\right)}^{2}dx=1-\int \sqrt{f_{X}\left(x\right)g_{X}\left(x\right)}\;dx. \end{array}$$

This gives a number between 0 and 1 where values closer to 0 represent more similarity between the distributions, whereas values close to 1 correspond to more different distributions.

We focus here on pairs of parameters (q,T) and (T,Q) for illustrative purposes, with distributions q∼Gamma(k,λ), T∼Gamma(ℓ,μ), and Q∼Gamma(r,γ) (when considered to be random). The aim is to study how randomness in these parameters translates into uncertainty in infection risk. In Figure A3, we plot the Hellinger distance between $f_{P_{infection}^{T,Q}}\left(p\right)$ and $f_{P_{infection}^{T}}\left(p\right)$ and between $f_{P_{infection}^{T,Q}}\left(p\right)$ and $f_{P_{infection}^{Q}}\left(p\right)$. In Figure A4, we plot the Hellinger distance between $f_{P_{infection}^{T,q}}\left(p\right)$ and $f_{P_{infection}^{T}}\left(p\right)$ and between $f_{P_{infection}^{T,q}}\left(p\right)$ and $f_{P_{infection}^{q}}\left(p\right)$. Both figures use the parameter regimes presented in Section 3.3.1. In both figures, for each Gamma‐distributed random parameter we set the mean parameter value to a constant while changing the shape parameter of their Gamma distribution (and accordingly changing the rate parameter so that the mean parameter value remains the same). Lighter areas in these plots indicate smaller Hellinger distances, indicating that the particular parameter being looked at (T in Figures A3a and A4a, q in Figure A4b and Q in Figure A3b) is the one that dominates the propagation of uncertainty into the per capita infection risk. Within each plot, in Figures A3 and A4, the patterns support the idea that a smaller shape (therefore an increased variance and positive skewness) for one random parameter, and a larger shape for another random parameter, leads to dominance on the impact on infection risk by the former over the latter.

In the case that one parameter has a small shape value, linearly increasing the shape of the other random parameter diminishingly increases the likeness of the first parameter's corresponding infection risk density to that of the case that both parameters are random (shown by a diminishingly decreasing Hellinger distance). For example, we see that in the case of random T and Q (Figure A3), when the shape of T is ℓ=2, there is not much difference in the similarity of $f_{P_{infection}^{T}}\left(p\right)$ (where the ventilation rate is considered constant and set to $\bar{Q}=r/\gamma )$ to $f_{P_{infection}^{T,Q}}\left(p\right)$ when the shape of Q varies between r=25 and r=50, indicated by unchanging color and lack of contour lines in that region of Figure A3a. On the other hand, as r increases, we see a noticeable increase in the Hellinger distance, $H(f_{P_{infection}^{T,Q}}\left(p\right),f_{P_{infection}^{Q}}\left(p\right))$, in Figure A3b (indicated by darker regions). A similar behavior can be seen when comparing Figure A4a and b. That is, for a small shape of T (e.g., ℓ=2), there is a negligible decrease in the Hellinger distance. One should expect that, when the shape of one parameter, say X, becomes increasingly large, then the infection risk density corresponding to the other parameter, $f_{P_{infection}^{Y}}\left(p\right)$, should tend toward the distribution in which both are random, $f_{P_{infection}^{X,Y}}\left(p\right)$. This is because, while technically still a Gamma‐distributed random parameter, X effectively becomes constant (random with asymptotically small variance).

The ranges of Hellinger distances are [0.012,0.704] for Figure A3 and [0.009,0.704] for Figure A4. The minute difference in the lower bounds could be due to the fact that having a Gamma‐distributed ventilation rate can lead to scenarios in which the rate is close to zero with positive probability, resulting in higher densities of high infection risk (as seen in Figure 10a,c), whereas this is not the case with Gamma‐distributed T or q where the same effect would require T or q to be significantly large.
